# Supplementary material for: Tripodal Pd metallenes mediated by Nb2C MXenes for boosting alkynes semihydrogenation
Source: Nat Commun. 2023 Feb 7;14:661. doi: 10.1038/s41467-023-36378-3 (PMC9905561; doi:10.1038/s41467-023-36378-3)
Supplement: Supplementary file 1 — Supplementary Information [file 41467_2023_36378_MOESM1_ESM.pdf]

Supplementary Information

Tripodal Pd metallenes mediated by Nb_2C MXenes for boosting alkynes semihydrogenation

Zhongzhe Wei[1, †], Zijiang Zhao[1, †], Chenglong Qiu[1], Songtao Huang[1], Zihao Yao[1, *], Mingxuan Wang[1], Yi Chen[1], Yue Lin[2], Xing Zhong[1], Xiaonian Li[1, *], Jianguo Wang[1, *]

[1] Institute of Industrial Catalysis, College of Chemical Engineering, Zhejiang University of Technology, Hangzhou 310032, P. R. China.

[2] Hefei National Research Center for Physical Sciences at the Microscale, University of Science and Technology of China, Hefei 230026, China.

[†] These authors contributed equally: Zhongzhe Wei, Zijiang Zhao.

* email: yaozihao@zjut.edu.cn; xnli@zjut.edu.cn; jgw@zjut.edu.cn.

Supplementary Methods

Synthesis of Cl-Nb_2C. Initially, 100 mL as-prepared Nb_2C solution (1.85 mg/mL) was added into 250 mL round bottom flask. Then, 20 mL HCl (36%) was added to the Nb_2C solution under magnetic stirring for 2 h at room temperature. Further, the precipitate was filtered and washed with double distilled water. Finally, the obtained solid precipitate was dried in a vacuum oven at 60 °C overnight.

Synthesis of Br-Nb_2C. Initially, 100 mL as-prepared Nb_2C solution (1.85 mg/mL) was added into 250 mL round bottom flask. Then, 20 mL HBr (48%) was added to the Nb_2C solution under magnetic stirring for 2 h at room temperature. Further, the precipitate was filtered and washed with double distilled water. Finally, the obtained solid precipitate was dried in a vacuum oven at 60 °C overnight.

Synthesis of O-Nb_2C. Initially, 100 mL as-prepared Nb_2C solution (1.85 mg/mL) was added into 250 mL round bottom flask. Then, 20 mL H_2O_2 (30%) was added to the Nb_2C solution under magnetic stirring for 1 h at room temperature. Further, the precipitate was filtered and washed with double distilled water. Finally, the obtained solid precipitate was dried in a vacuum oven at 60 °C overnight.

Synthesis of Pd/Cl-Nb_2C, Pd/Br-Nb_2C and Pd/O-Nb_2C. Firstly, 150 mg as-prepared Cl-Nb_2C, Br-Nb_2C and O-Nb_2C powder was added into 100 mL round bottom flask. Then, 50 mL deionized water was added to the Cl-Nb_2C, Br-Nb_2C and O-Nb_2C powder under magnetic stirring for 30 min at room temperature. Further, 1.25 mL of $PdCl_2$ (1 mg/mL) was added to the Cl-Nb_2C, Br-Nb_2C and O-Nb_2C solution under magnetic stirring for 4 h at 35 °C, respectively. The precipitate was filtered and washed with double distilled water. Finally, the obtained solid precipitate was dried in a vacuum oven at 60 °C overnight.

Synthesis of Pd/Al_2O_3. Initially, 200 mg γ-Al_2O_3 was dispersed into 50 mL deionized water in the bottom flask. Then, 1.7 mL of 1 mg/mL $PdCl_2$ aqueous solution was added to the mixture of γ-Al_2O_3 solution under magnetic stirring for 10 min at 25 °C. Further, 10 mL $NaBH_4$ aqueous solution (20 mg/mL) was added into above solution dropwise. Next, the 0.5% Pd/Al_2O_3 precipitate was filtered and washed with double distilled water. Finally, the obtained solid precipitate was dried in a vacuum oven at 60 °C overnight.

Synthesis of Pd/TiO_2. Initially, 200 mg P25-TiO_2 was dispersed into 50 mL deionized water in the bottom flask. Then, 1.7 mL of 1 mg/mL $PdCl_2$ aqueous solution was added to the mixture of P25-TiO_2 solution under magnetic stirring for 10 min at 25 °C. Further, 10 ml $NaBH_4$ aqueous solution (20 mg/mL) was added into above solution dropwise. Next, the 0.5% Pd/TiO_2 precipitate was filtered and washed with double distilled water. Finally, the obtained solid precipitate was dried in a vacuum oven at 60 °C overnight.

Computational Details

1. Parametrization Method for the Interatomic Potential. To fit the force field parameters, DFT calculations with periodic boundary conditions were performed using the VASP code. And the fitting data was based on structures and interaction energies of Pd_4 cluster supported on MXenes (Br_2Nb_2C, Cl_2Nb_2C, O_2Nb_2C and Nb_2C) with a 3 × 3 × 1 unit cell. The interaction energy is defined as

$$E_{\text{int}} = E_{\text{Pd}_4/\text{sub}} - E_{\text{Pd}_4} - E_{\text{sub}} \quad (1)$$

where $E_{\text{Pd}_4/\text{sub}}$, E_{Pd_4} and E_{sub} is total energy of 4-atom Pd cluster on substrate, 4-atom Pd cluster and substrate, respectively.

First, the four-atom Pd cluster supported on substrates were optimized by DFT calculations. Then the DFT-optimized cluster placed on the surface of substrate at a distance R without structure reoptimization, where R was varied between 1 and 6.0 Å at intervals of 0.1 Å. And a total of 36 structures were selected for each system to fit the force field parameters.

During the fitting process, the interaction energy is the sum of all the Pd-M interactions, where M indicates each atom in the substrate with a distance smaller than 8 Å from the Pd atoms. To obtain accurate parameters effectively, we employed Levenberg-Marquardt (LM) method[1,2] to solve the Morse potential function, and the function of convergence criterion for fitting was defined as

$$e = \sum_R (E_{\text{fit}} - E_{\text{DFT}})^2 \quad (2)$$

where R represents the distance between the four-atom Pd cluster and the surface of substrate. E_{fit} and E_{DFT} are the interaction energies obtained by fitting results and DFT calculations, respectively.

2. MD simulations. All MD simulations were performed using the large-scale

atomic/molecular massively parallel simulator (LAMMPS) software[3]. Pd_{561} nanoparticles with the structure of cuboctahedron was selected as supported metal models. Nb_2C and its derivative (Cl_2Nb_2C, Br_2Nb_2C and O_2Nb_2C) were selected as the substrates, whose structures were optimized by DFT calculations.

In MD simulation, the embedded-atom method (EAM) was adopted to model metal–metal interactions[4,5]. And the Morse potential was employed for describing the interaction between the Pd atom and substrate, which was obtained by fitting results of the DFT calculations.

The MD simulation was performed in a NVT ensemble with a time step of 2.5 fs. The equilibrium structure of supported nanoparticle was obtained by annealing process. First, the initial structure was optimized at 300 K for 50 ps. Then, the nanoparticle was heated to the melting point and equilibrated at 1400 K for 50 ps. Finally, the nanoparticle was cooled to 300 K. In MD simulation, the substrate layers were fixed, and the periodic boundary conditions were applied parallel to the substrates (x and y directions), while a non-periodic boundary condition was applied perpendicular to the substrates (z direction).

3. DFT Computational Methods. To study CHCR hydrogenation, two types of periodic surfaces were selected in our simulation, namely, Pd(111) and two layers Pd(111)/Nb_2C. The p(4x4) unit cell was utilized for Pd(111) and two layers Pd(111)/Nb_2C. Both models were built with a 15 Å vacuum region to avoid the neighboring slab interactions. The thickness of the surfaces was verified to be sufficient. All DFT calculations were performed with generalized gradient approximation (GGA) of the Perdew-Burke-Ernzerhof (PBE)[6] functional using the VASP simulation package[7,8]. The approach of project-augmented-wave (PAW) was exploited to describe the interaction between core-electron and valence electron[9,10]. A plane-wave basis set was expanded with the kinetic cutoff energy of 400 eV on valence electronic states. The Brillouin zone samplings of k-point values were tested using CHCR chemisorption energy, and a k-point sampling of 3x3x1 was adopted with the convergence value less than 0.1 eV. The effect of vdW interaction is significant for the reaction mechanism in our previous study[11-15]. Therefore, the DFT-D3 method[16-19] of Grimme et al. was utilized to calculate all the energetics and structures of the intermediates and transition

states. The energetics and structures were calculated using the conjugate-gradient algorithm until the Hellman Feynman forces on each atom less than 0.01 eV/Å. The transition states (TSs) were searched using the method called a constrained optimization scheme[20-23]. The transition states were confirmed by two rules: (i) all forces on atoms have been optimized to be less than 0.01 eV/Å; (ii) the total energy is a maximum along the reaction coordinate but a minimum with respect to all other degrees of freedom. All the optimized initial states, transition states and final states were verified by frequency analysis using a finite displacement method to the mass-weighted Hessian matrix[24]. Vibrational frequency analyses were performed to confirm the integrity of initial states, transition states and final states. The zero-point energy (ZPE) correction was calculated as follows:

$$ZPE = \sum_i \frac{h\nu_i}{2} \qquad (1)$$

where h is Planck's constant and the standard molar vibrational internal energy contribution is calculated as follows:

$$U^o_{vib} = RT\sum_i \frac{h\nu_i/K_B}{e^{h\nu_i/K_BT}-1} \qquad (2)$$

where K_B is the Boltzmann constant and R is the gas constant. The standard molar vibrational entropy is given by:

$$S^O_{vib} = R\sum_i [\frac{\frac{h\nu_i}{K_BT}}{e^{\frac{h\nu_i}{K_BT}}-1} - \ln(1-e^{-h\nu_i/K_BT})] \qquad (3)$$

Overall, adding the free energy corrections together, the standard molar Gibbs free energy change for the elementary reaction for the hydrogenation reaction can be written as follow:

$$\Delta G^0 = \Delta E + \Delta ZPE + \gamma RT\left(1+\ln\frac{P}{P_0}\right) + \Delta U^0 - T\Delta S^0 \qquad (4)$$

where ΔE represents the difference of the total energies from the VASP calculation. If it is gaseous molecule, then 1 is chosen as the value of γ and 0 is selected for γ for surface reactant and P is the partial pressure.

The free-energy corrections for molecules in the gas phase were calculated using the Gaussian 09 package[13], and the basis set was B3LYP/6-311g[14,15].

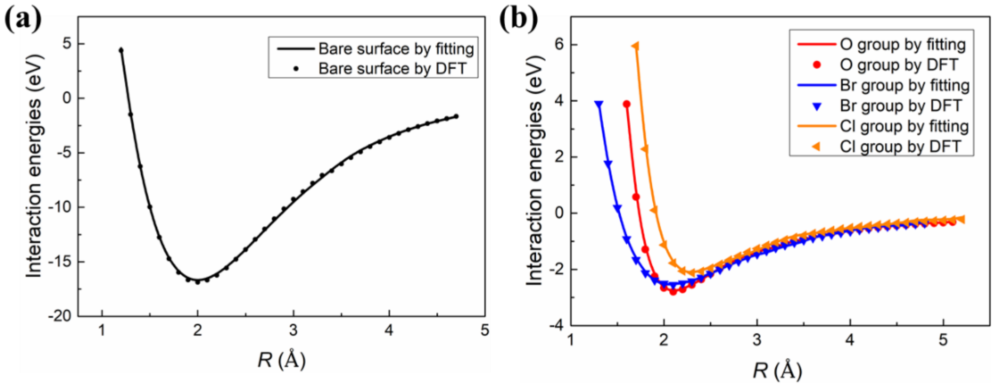

Supplementary Fig. 1 | Interaction energies for the DFT calculations and the fitted Morse potential curve with the structures of (a) Pd_4/Nb_2C, (b) Pd_4/O_2Nb_2C, Pd_4/Br_2Nb_2C and Pd_4/Cl_2Nb_2C.

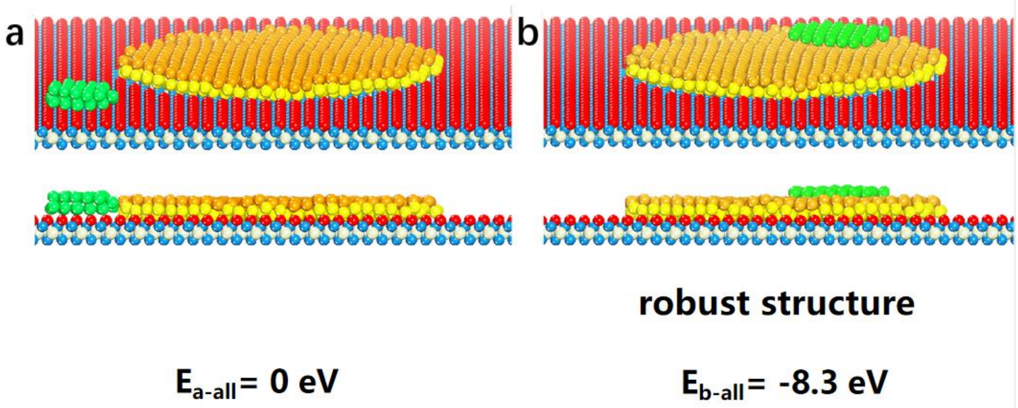

Supplementary Fig. 2 | Snapshots and energies of Pd_{923} nanoparticles supported on substrate of Nb_2C surrounded by O group for different sites, 923 atoms refer to all Pd atoms on Nb_2C, including green, yellow and orange Pd atoms. (a) green Pd clusters anchor on the Nb_2C surrounded by O group. (b) green Pd atoms accumulate as layer on the Pd metallenes.

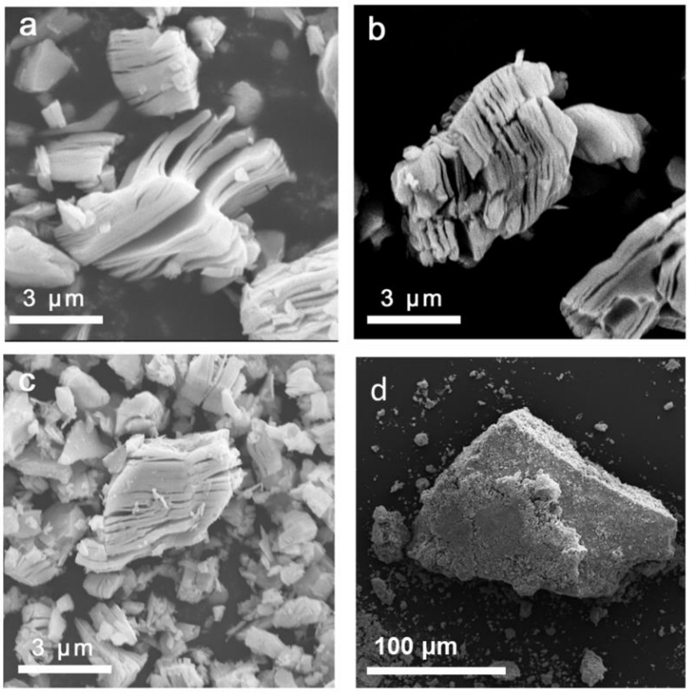

Supplementary Fig. 3 | SEM images of (a) Pd/Nb_2C, (b) Pd/Cl-Nb_2C, (c) Pd/Br-Nb_2C, (d) Pd/O-Nb_2C, respectively.

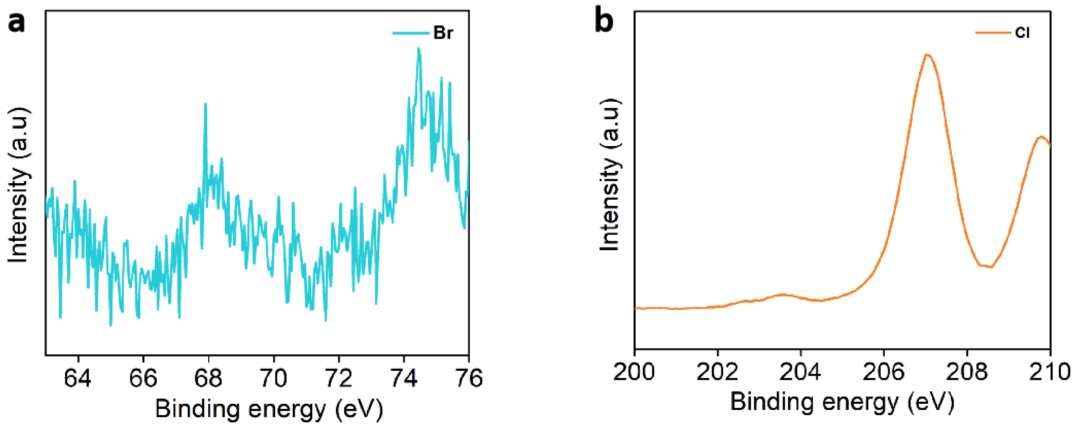

Supplementary Fig. 4 | High resolution XPS spectrum of Pd/Br-Nb_2C and Pd/Cl-Nb_2C. (a) Br 2*p*; (b) Cl 3*d*.

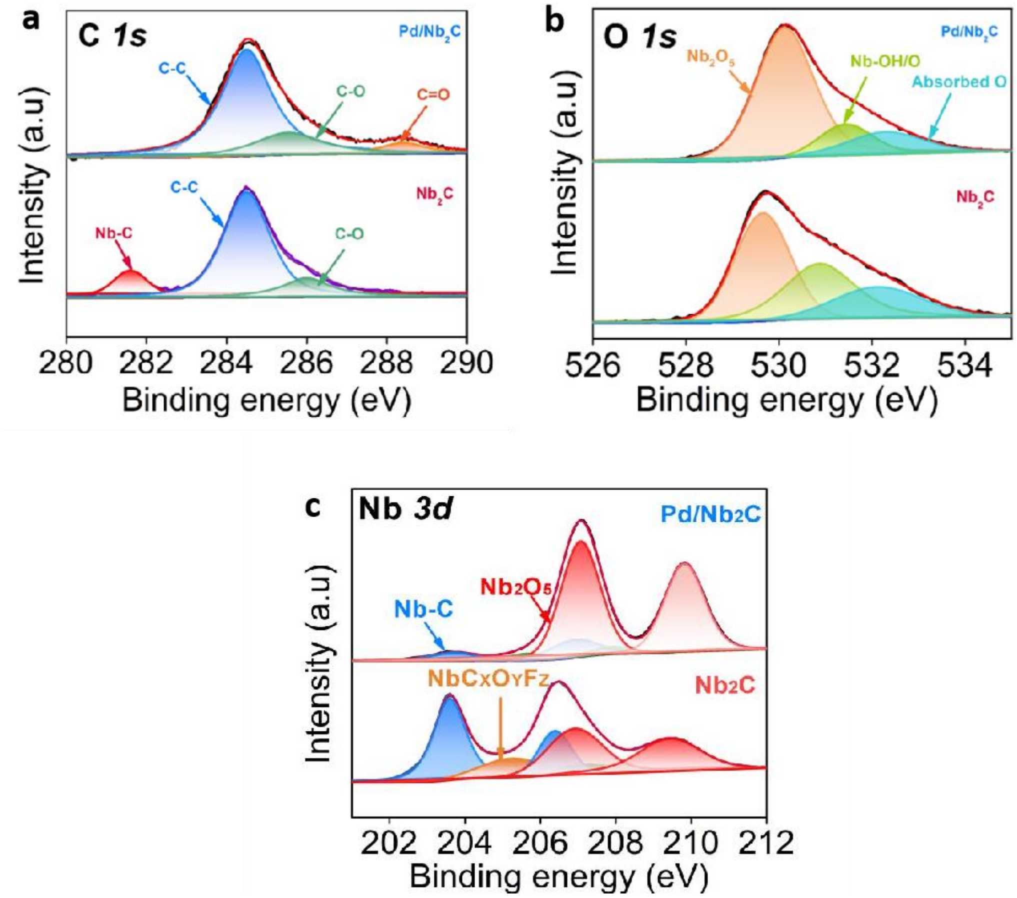

Supplementary Fig. 5 | High resolution XPS spectra of Pd/Nb_2C and Nb_2C. (a) C 1*s*; (b) O 1*s*; (c) Nb 3*d*.

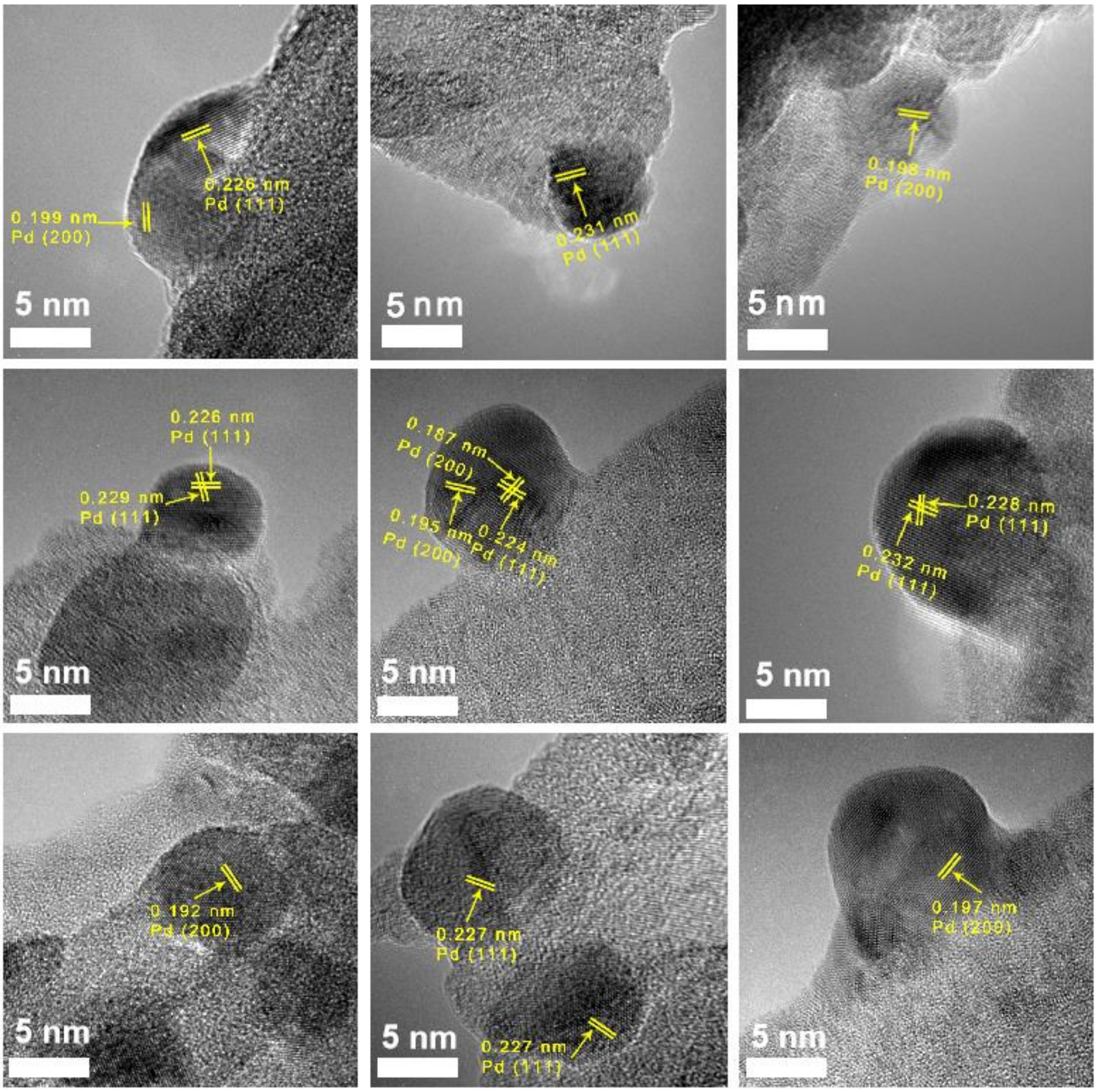

Supplementary Fig. 6 | HRTEM images of Pd/Cl-Nb_2C.

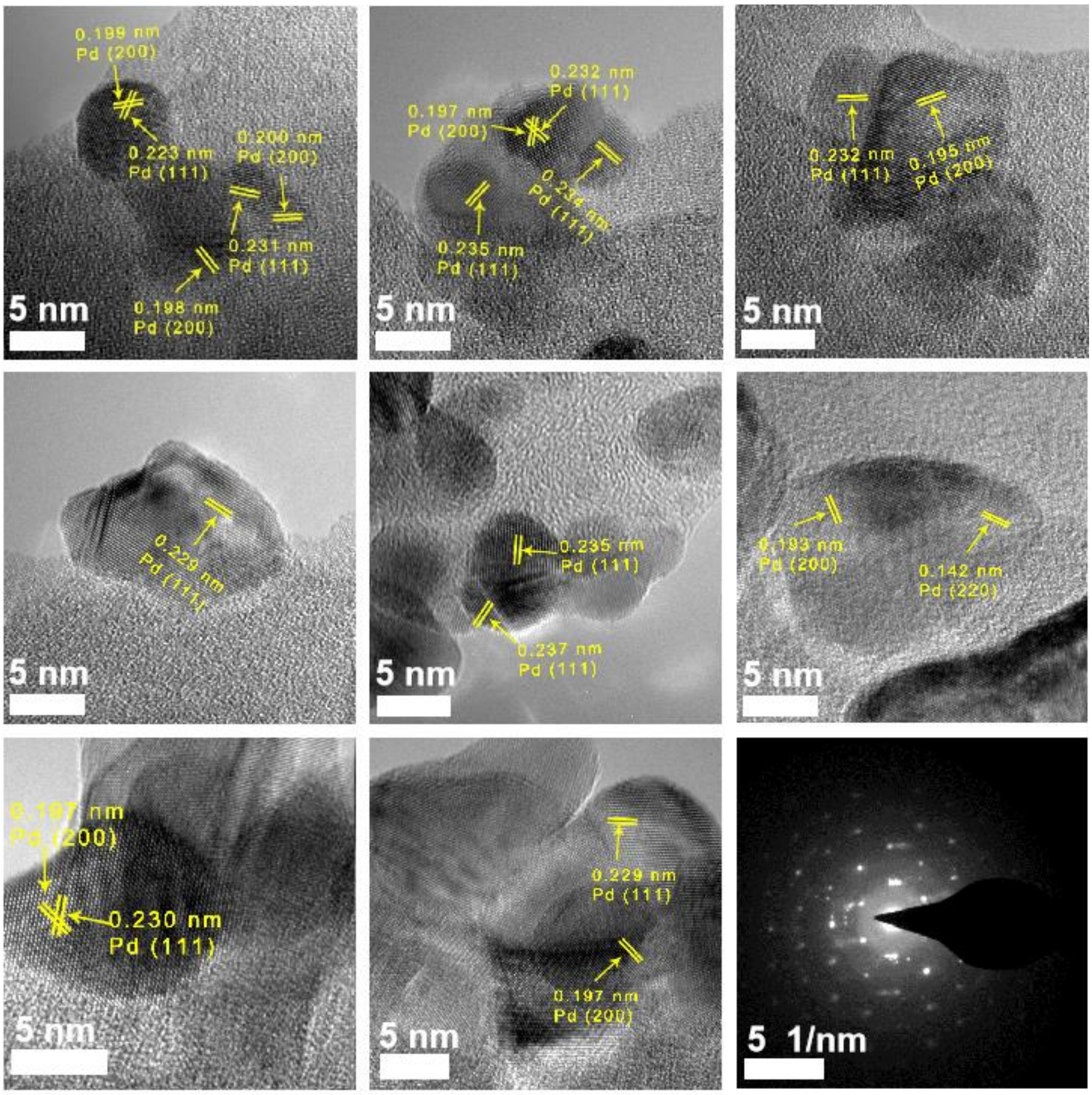

Supplementary Fig. 7 | HRTEM images and selected area electron diffraction (SAED) pattern of Pd/Br-Nb_2C.

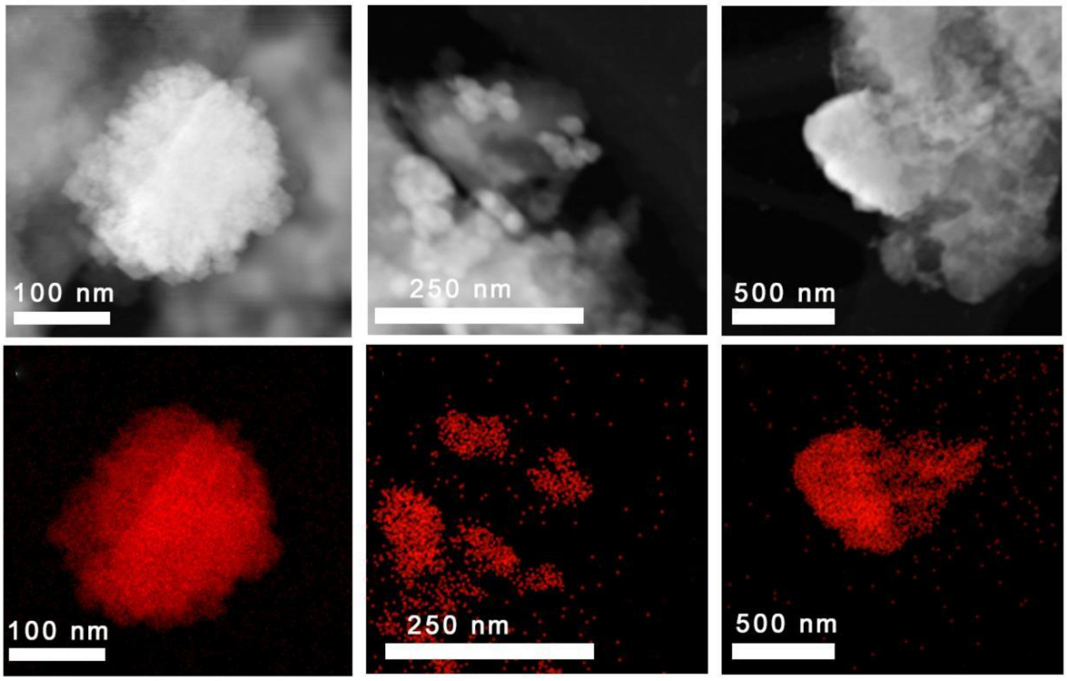

Supplementary Fig. 8 | TEM images and elemental mapping of Pd/O-Nb_2C.

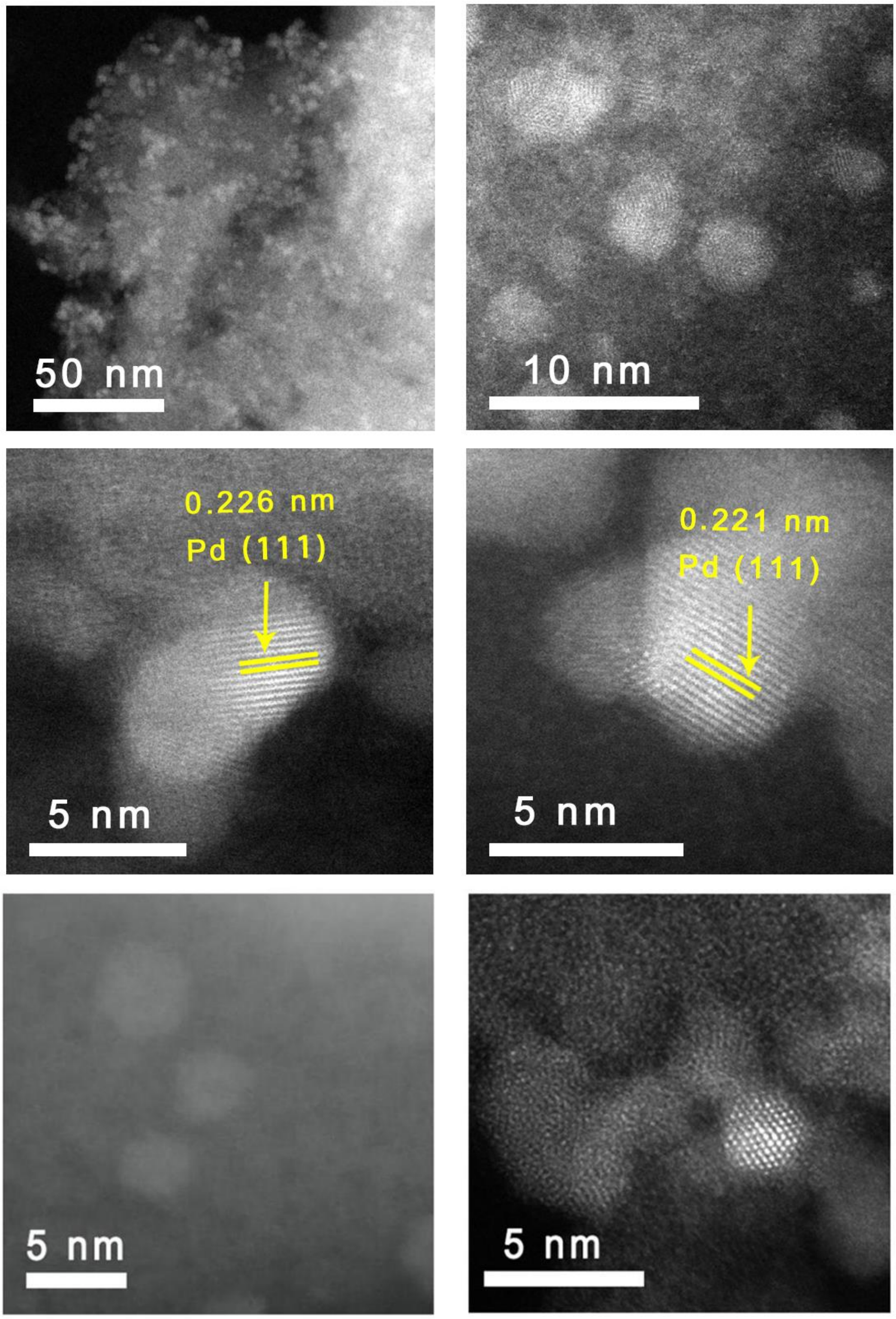

Supplementary Fig. 9 | HAADF-STEM images of Pd/Nb_2C.

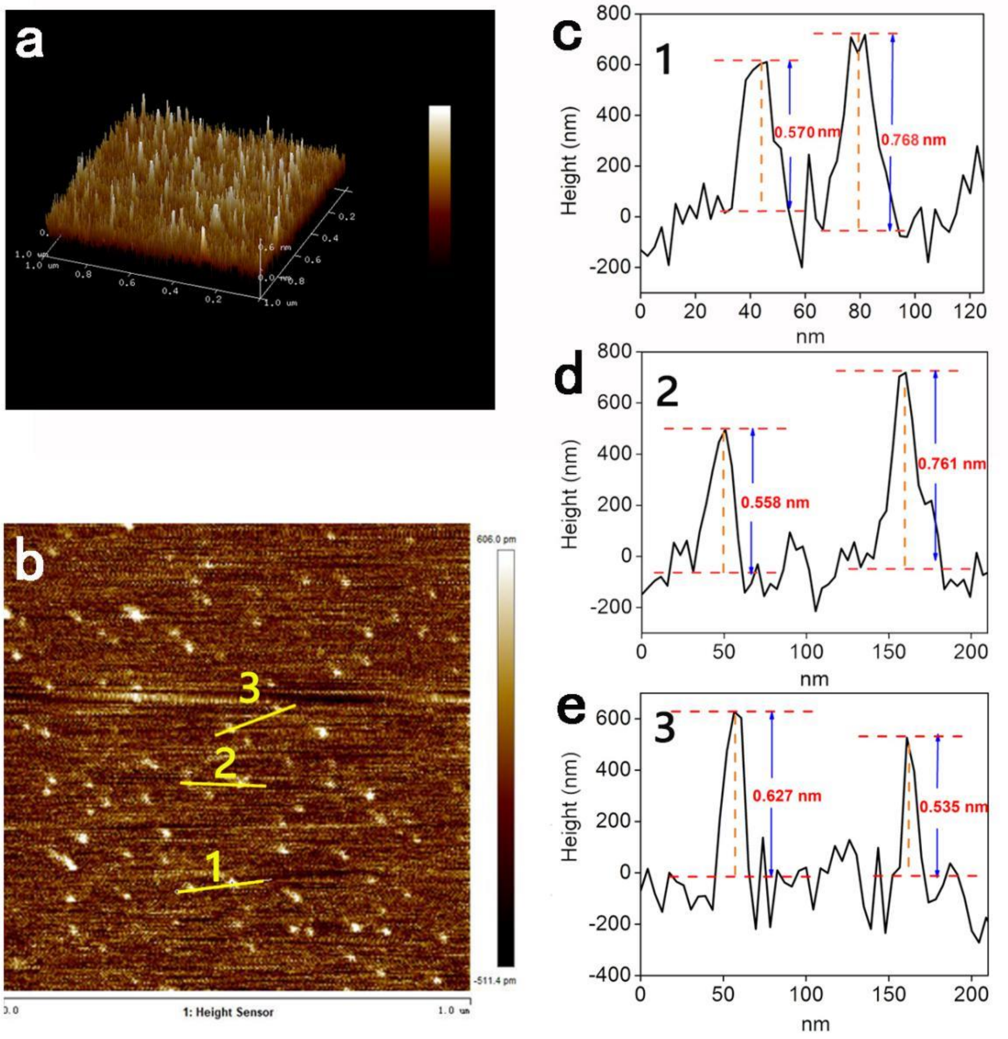

Supplementary Fig. 10 | AFM images of Pd/Nb_2C. (a) 3D tapping mode AFM image of Pd metallenes. (b) 2D AFM image of Pd/Nb_2C corresponding to the tapping mode AFM image. (c, d, e) Height profiles along the three marked lines in (b).

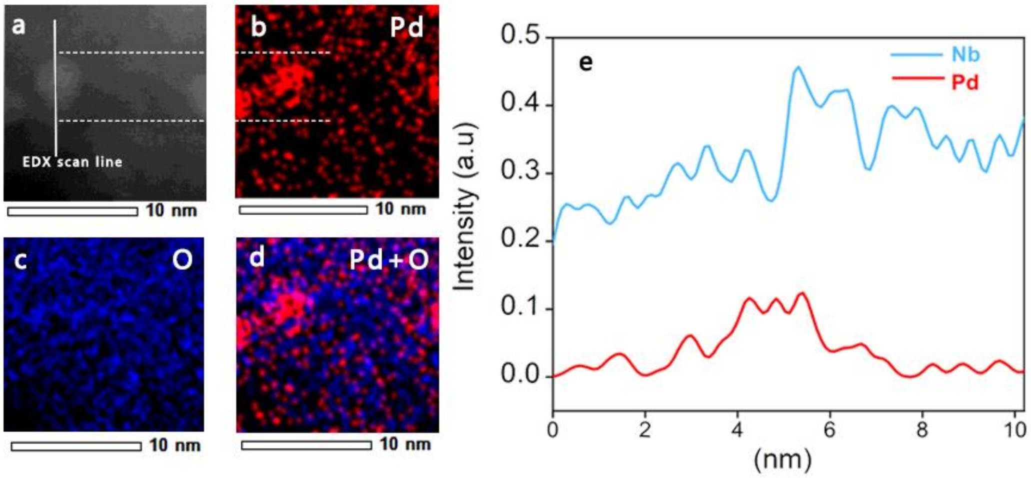

Supplementary Fig. 11 | Elemental mappings of Pd/Nb_2C and EDX scan line. (a) HAADF-STEM image of Pd/Nb_2C. Elemental mapping of (b) Pd, (c) O and (d) composite mapping of Pd vs O. (e) Integrated pixel intensities of Pd (red) and Nb (blue) in Pd/Nb_2C.

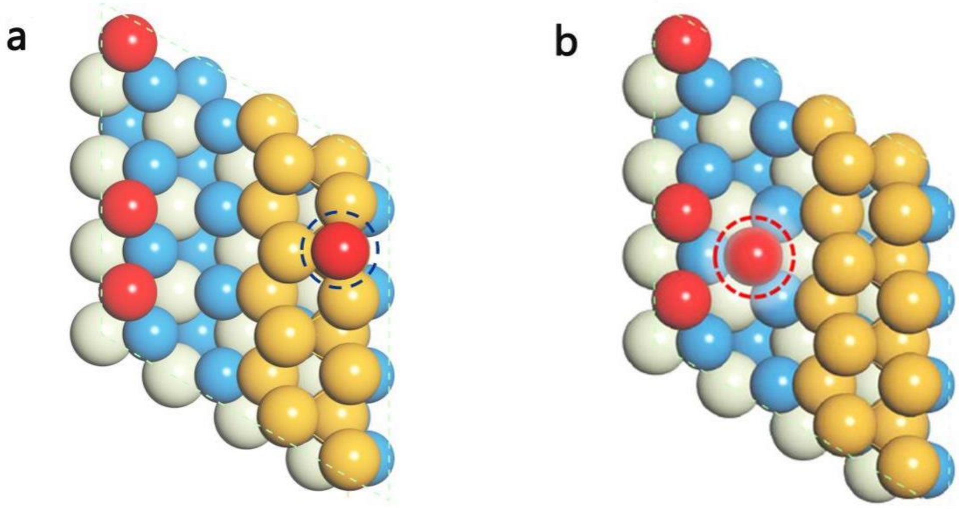

Supplementary Fig. 12 | Two DFT models of Pd/Nb_2C. (a) O atom on the surface of Pd in Pd/Nb_2C. (b) O atom at the interface of Pd and Nb_2C.

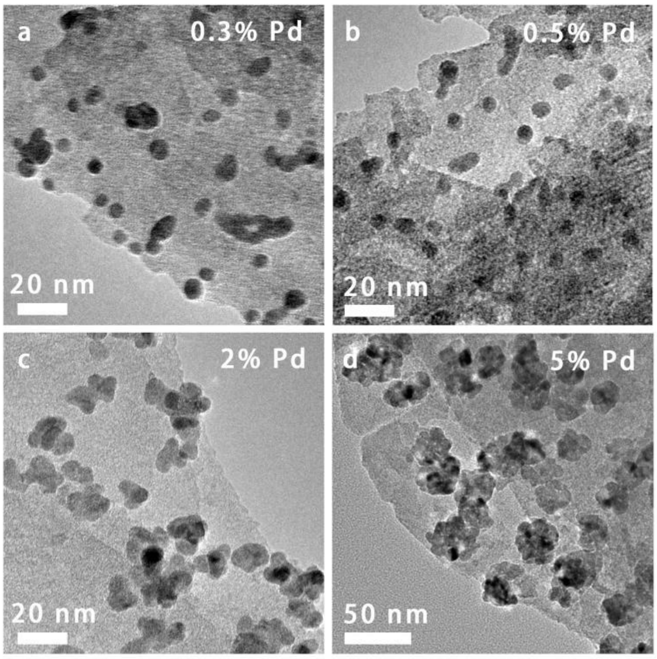

Supplementary Fig. 13 | TEM images of Pd/Nb_2C samples with different Pd loadings. (a) 0.3% Pd/Nb_2C; (b) 0.5% Pd/Nb_2C; (c) 2% Pd/Nb_2C; (d) 5% Pd/Nb_2C.

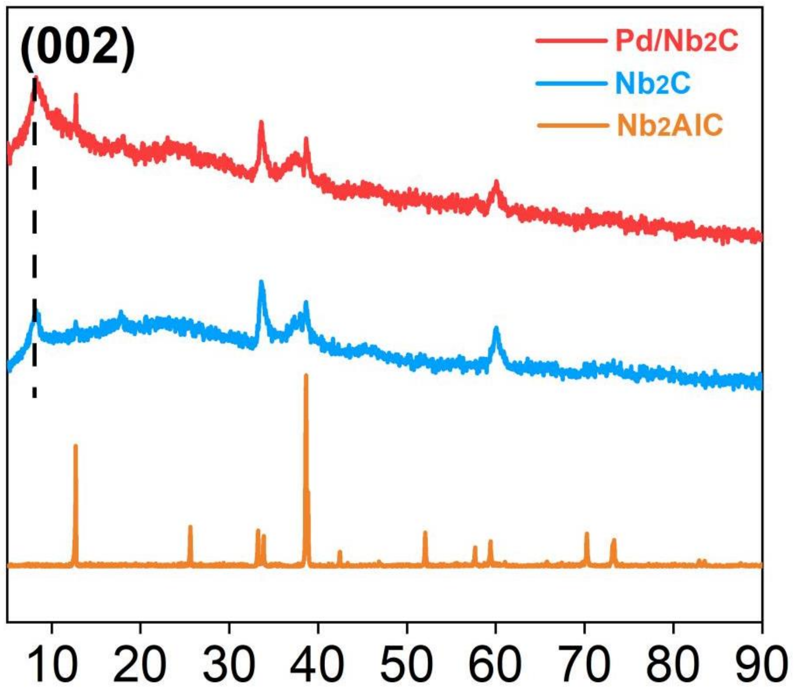

Supplementary Fig. 14 | XRD patterns of the Pd/Nb_2C, Nb_2C and Nb_2AlC.

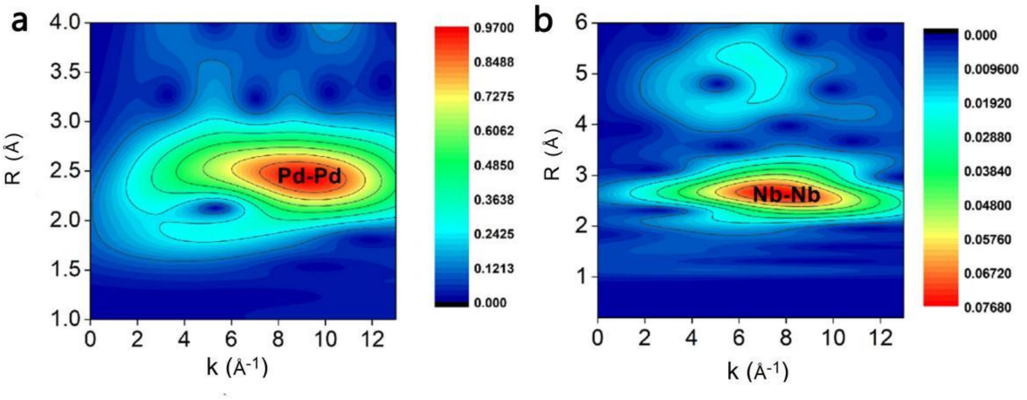

Supplementary Fig. 15 | WT-EXAFS plots of Pd in the Pd foil and WT-EXAFS plots of Nb in the Nb foil. (a) Pd; (b) Nb.

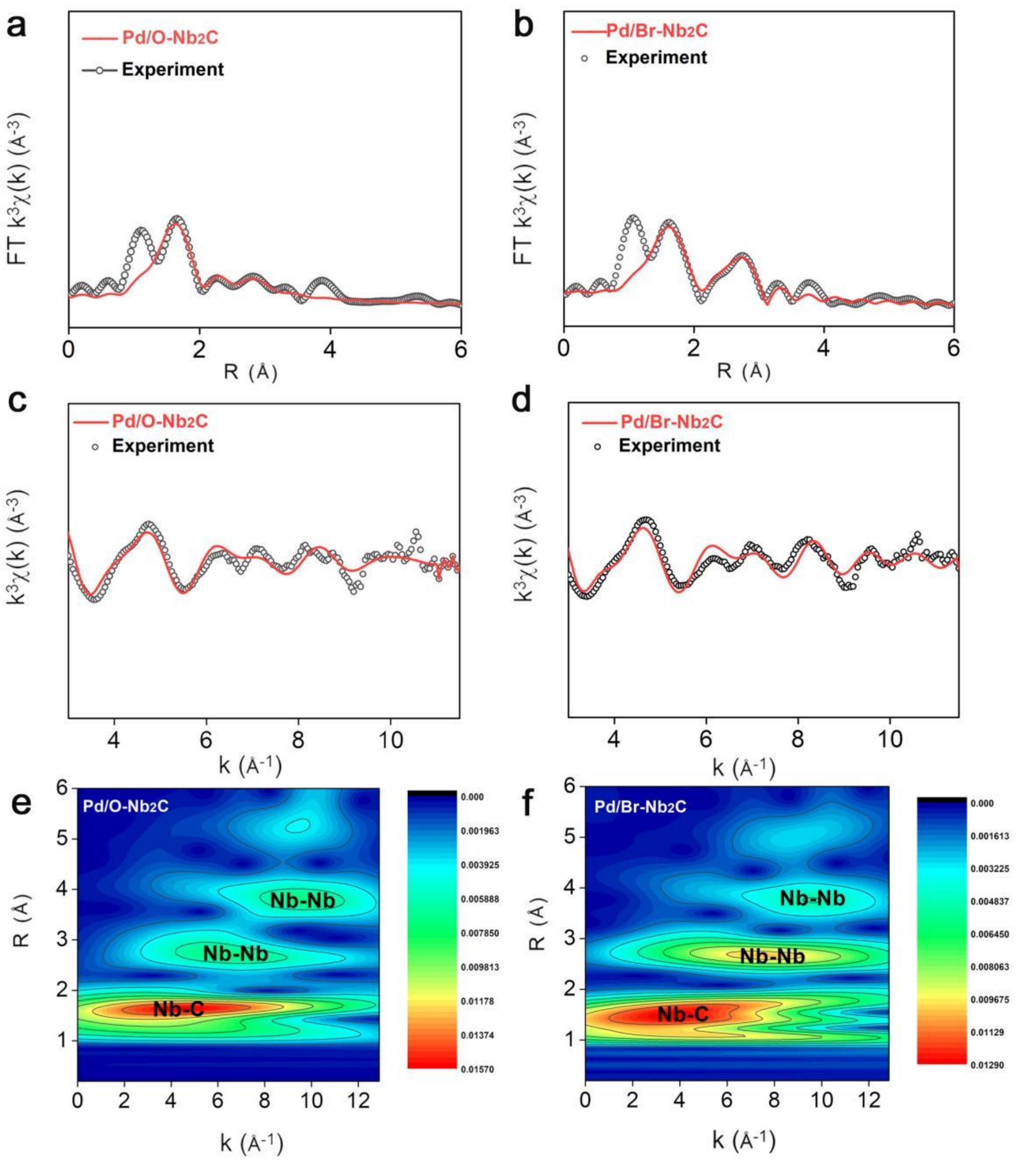

Supplementary Fig. 16 | Fourier transform spectra of Nb *K*-edge EXAFS for Pd/O-Nb_2C and Pd/Br-Nb_2C. (a, c, e) XAFS analysis of Pd/O-Nb_2C, (a) R-spaced FT-EXAFS of Nb *K*-edge; (c) k-space; (e) WT-EXAFS plots of Nb. (b, d, f) XAFS analysis of Pd/Br-Nb_2C, (b) R-spaced FT-EXAFS of Nb *K*-edge; (d) k-space; (f) WT-EXAFS plots of Nb.

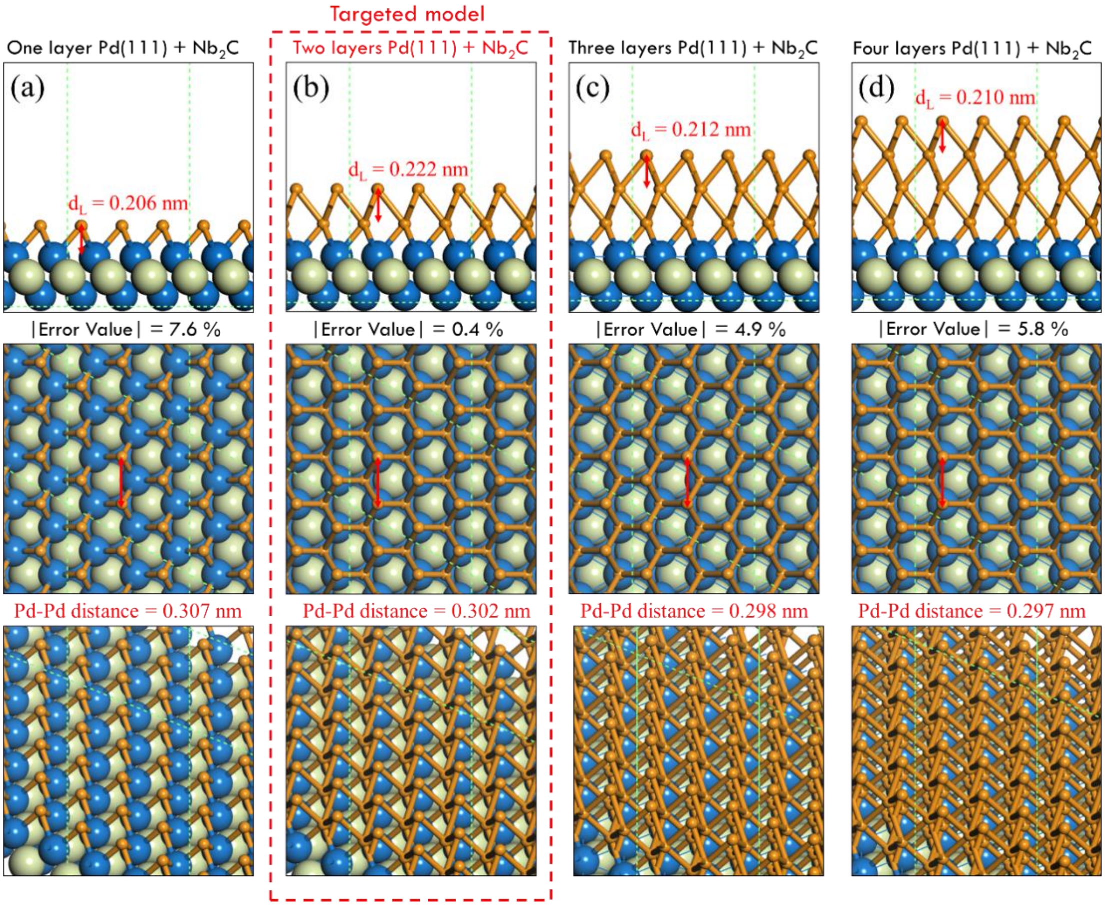

Supplementary Fig. 17 | The side view (up), top view (middle) and oblique view (bottom) of the simulated surfaces. (a) One layer Pd(111)+Nb_2C; (b) Two layers Pd(111)+Nb_2C; (c) Three layers Pd(111)+Nb_2C; (d) Four layers Pd(111)+Nb_2C. The d_L represents simulated lattice space. $|Error\ value| = \left|\frac{experiment\ value - d_L}{experiment\ value}\right|$, experiment value = 0.223 nm.

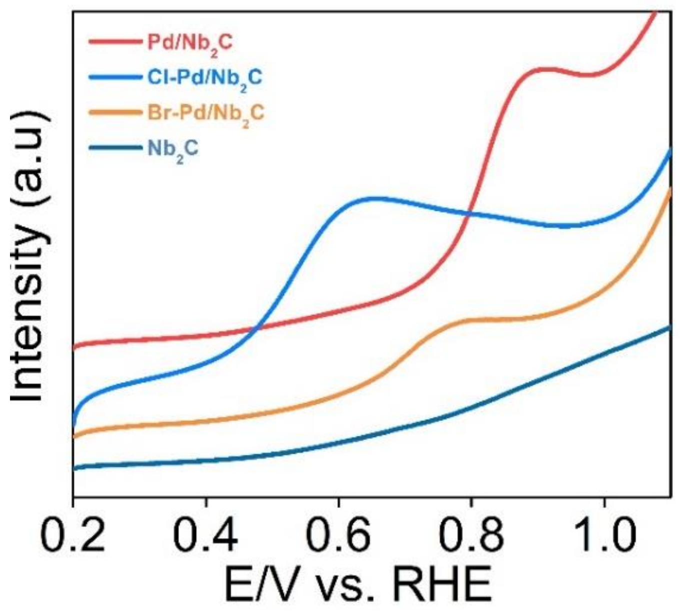

Supplementary Fig. 18 | CO stripping voltammetry curves of Nb_2C, Pd/Nb_2C, Pd/Cl-Nb_2C, and Pd/Br-Nb_2C.

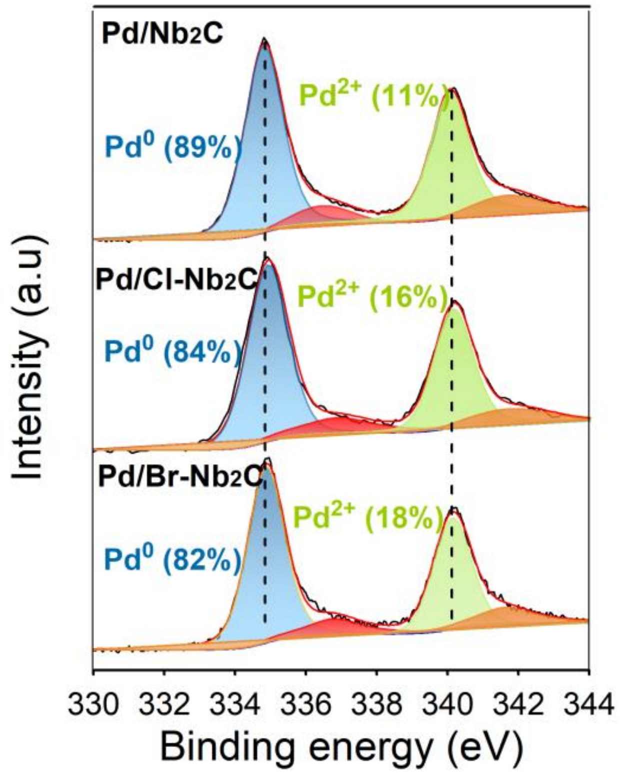

Supplementary Fig. 19 | Pd 3*d* XPS spectra of Pd/Nb_2C, $Pd/Cl\text{-}Nb_2C$, and $Pd/Br\text{-}Nb_2C$.

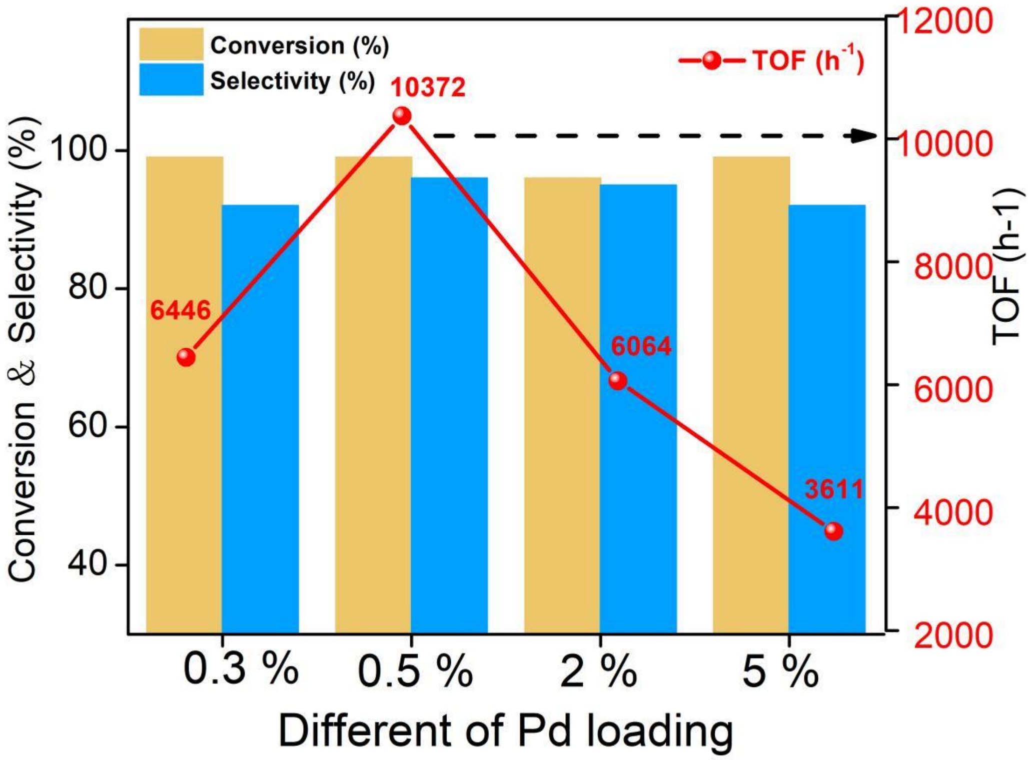

Supplementary Fig. 20 | Catalytic performance of Pd/Nb_2C with different Pd loadings. Reaction conditions: 2 mmol of phenylacetylene, 10 mg of catalysts (0.014 mol %, 0.023 mol %, 0.092 mol % and 0.23 mol % Pd for 0.3% Pd/Nb_2C, 0.5% Pd/Nb_2C, 2% Pd/Nb_2C and 5% Pd/Nb_2C), 5 mL of ethanol, 298 K, 0.1 MPa of H_2. The catalytic behaviors of Pd/Nb_2C with different Pd loadings from 0.3 wt. % to 5 wt. % were evaluated by the hydrogenation of phenylacetylene, and these reactions were completed within 55 min, 40 min, 12 min, and 7 min, respectively.

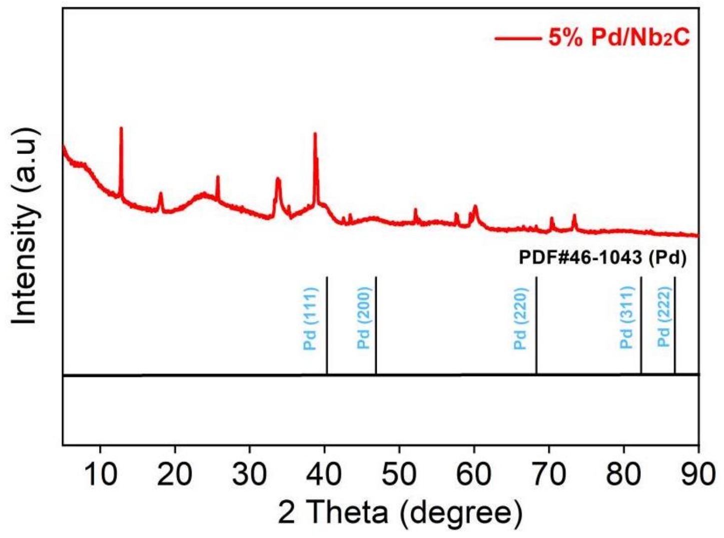

Supplementary Fig. 21 | XRD pattern of 5% Pd/Nb_2C.

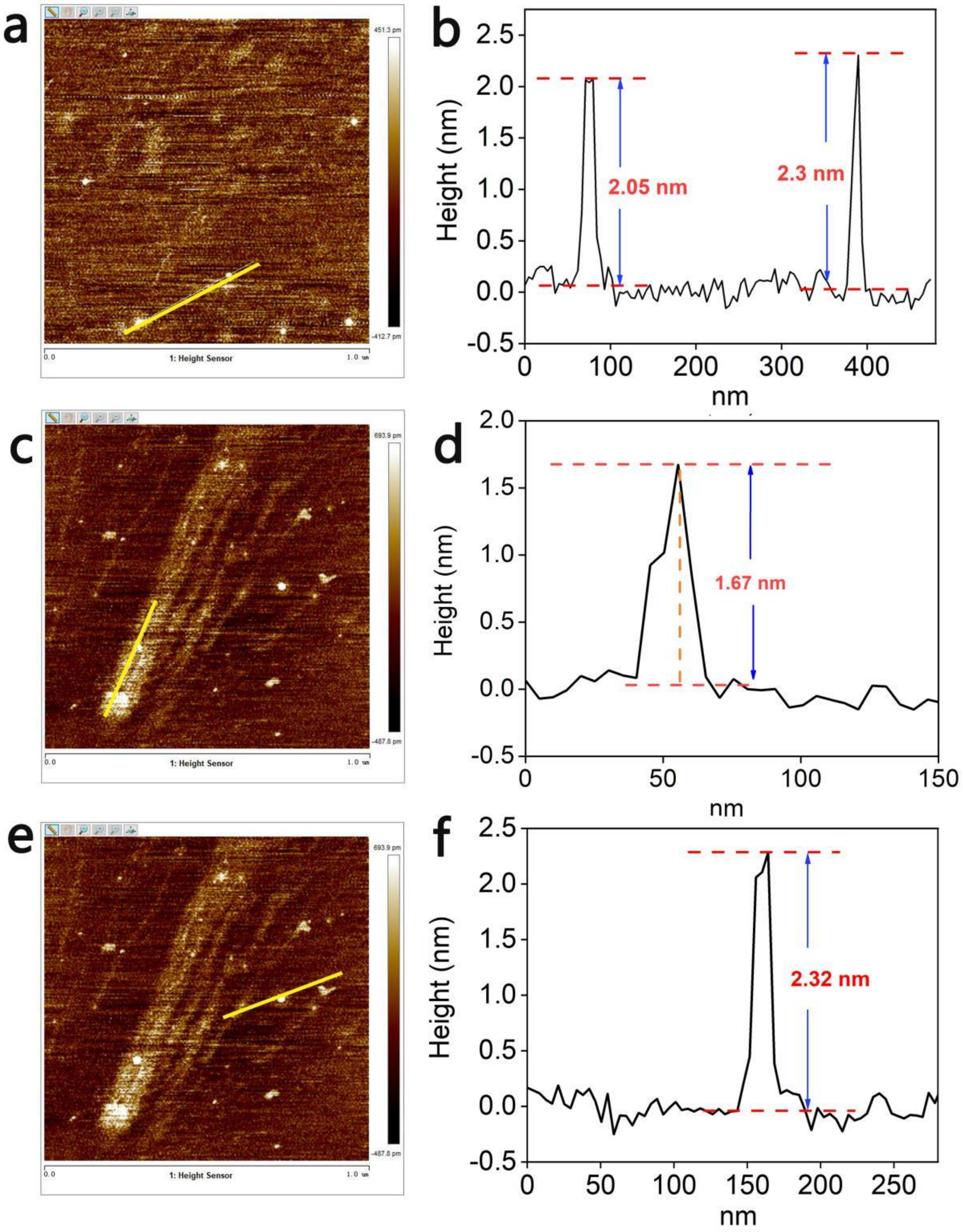

Supplementary Fig. 22 |AFM images of 5% Pd/Nb_2C. (a, c, e) 2D AFM images of 5% Pd/Nb_2C. (b, d, f) Height profiles along the marked line in (a), (c) and (e).

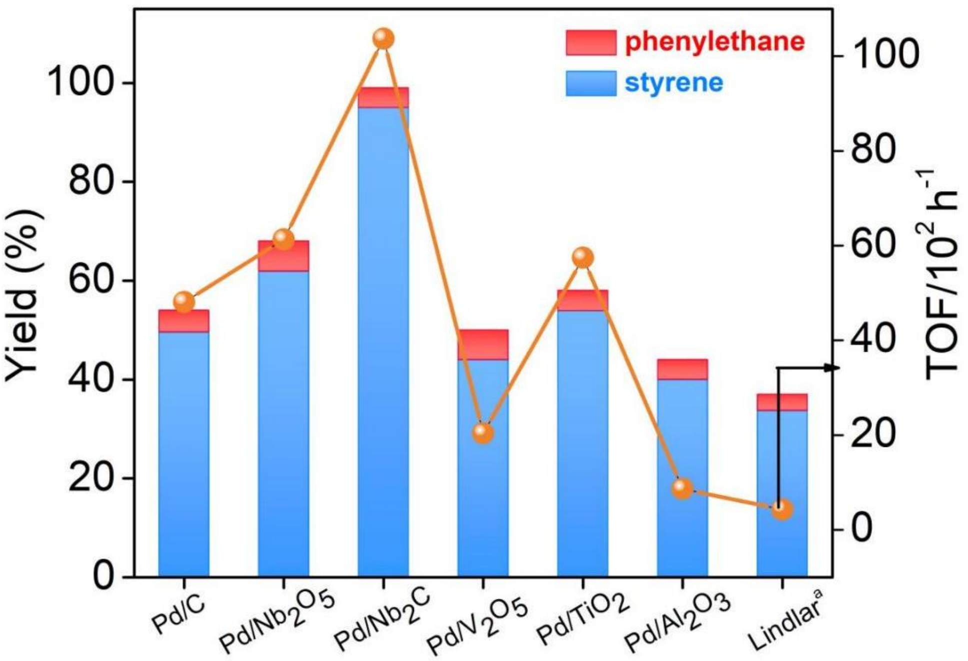

Supplementary Fig. 23 | TOF values and the yields of products for various supported Pd catalysts. Conditions: 2 mmol of phenylacetylene, 10 mg of catalyst with 0.5 wt. % Pd (0.023 mol % Pd), 5 mL of ethanol, 298 K, 0.1 MPa of H_2, 40 min. [a] The dosage of Lindlar catalyst (5 wt. % Pd loaded on $CaCO_3$ with Pb poisoning) was 10 mg (0.23 mol % Pd).

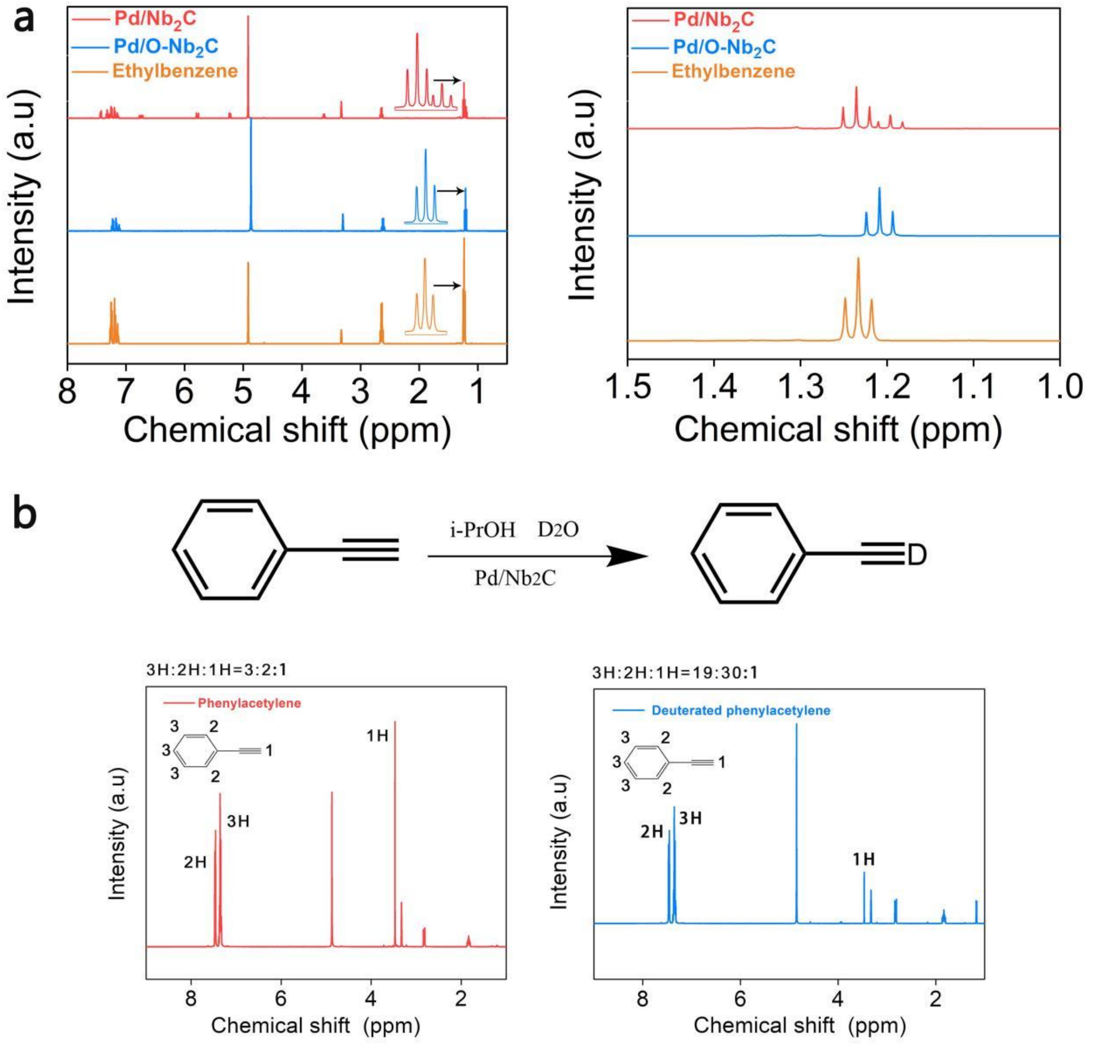

Supplementary Fig. 24 | NMR data for deuterated phenylacetylene catalyzed by Pd/Nb_2C. NMR data for the products of deuterated phenylacetylene carried out by using Pd/Nb_2C in i-PrOH as the solvent, and D_2O as deuterium source. These results also confirmed the existence of hydrogen proton in the reaction mechanism for Pd/Nb_2C. (a) Chemical shift of 1-1.5 ppm in **Fig. 5e** was amplified. (b) Deuterization reaction diagram of phenylacetylene and deuterization verification. (Red) Phenylacetylene, the peaks around 3.47 ppm were corresponding to H atoms on carbon-carbon triple bond. (Blue) Deuterated phenylacetylene, the peaks around 3.46 ppm decreased in the intensity compared to Red.

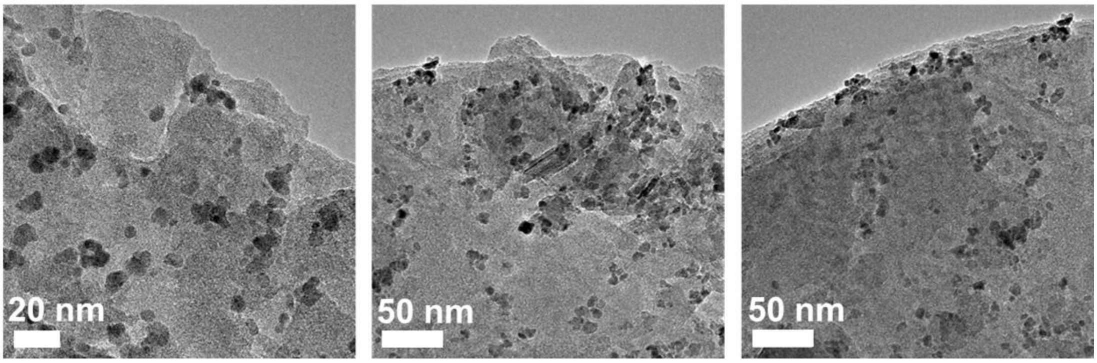

Supplementary Fig. 25 | TEM images of the used Pd/Nb_2C.

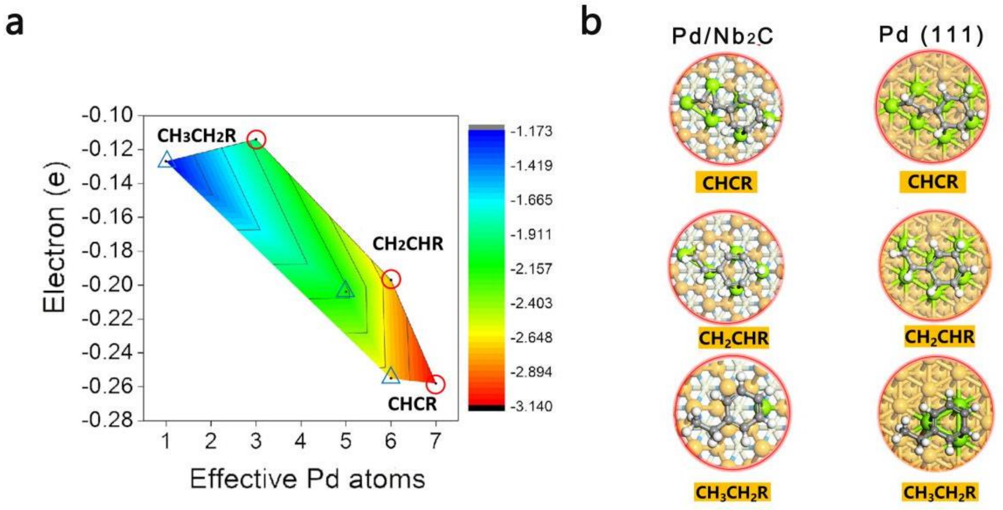

Supplementary Fig. 26 | (a) 2-D heat map describing the chemisorption energy of different catalysts as a function of effective Pd atoms and Bader charge in molecules. The red circle means adsorbate on Pd (111). The blue triangle represents adsorbate on Pd/Nb_2C. (b) The left row represents a top view of CHCR, CH_2CHR, and CH_3CH_2R on Pd/Nb_2C. The right row represents a top view of CHCR, CH_2CHR and CH_3CH_2R on Pd (111). The atoms in green represent effective Pd atoms. Herein, the effective Pd atoms represent the number of Pd atoms directly bonded with the molecules.

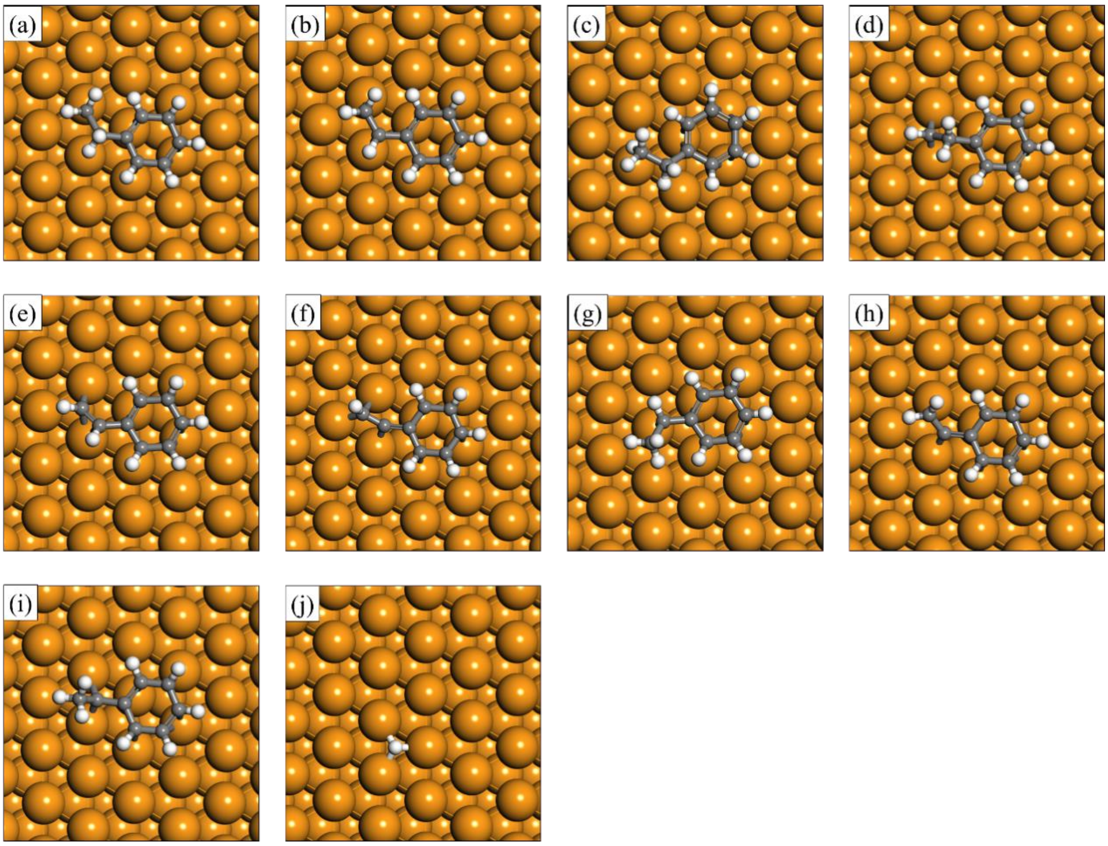

Supplementary Fig. 27 | Top views of all the optimized geometries of intermediates on Pd (111) for the hydrogenation of phenylacetylene. (a) CH_2CH_2R; (b) CH_2CHR; (c) CH_3CH_2R; (d) $CHCH_2R$; (e) CHCHR; (f) CHCR; (g) $CHRCH_3$; (h) $CRCH_2$; (i) $CRCH_3$; (j) H.

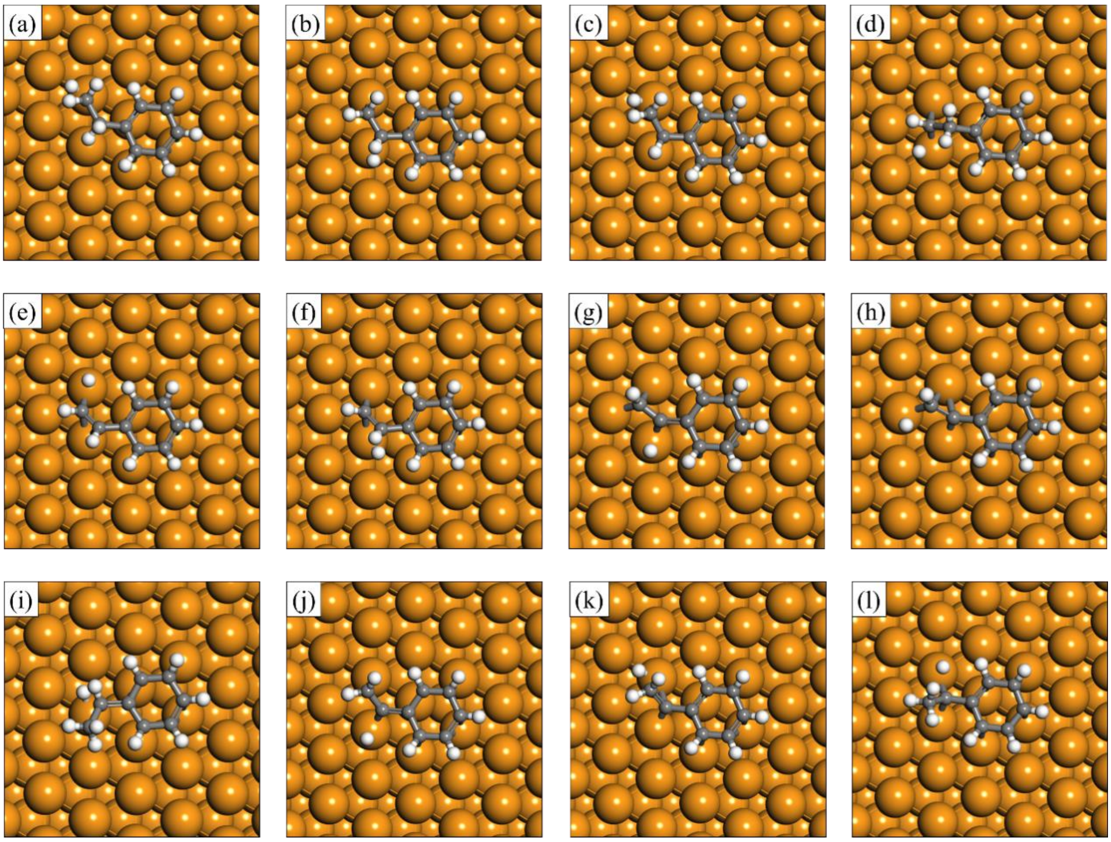

Supplementary Fig. 28 | Top views of calculated transition state geometries on Pd (111) for the hydrogenation of phenylacetylene. (a) $CH_2CH_2R+H \rightleftarrows CH_3CH_2R$; (b) $CH_2CHR+H \rightleftarrows CH_2CH_2R$; (c) $CH_2CHR+H \rightleftarrows CHRCH_3$; (d) $CHCH_2R+H \rightleftarrows CH_2CH_2R$; (e) $CHCHR+H \rightleftarrows CH_2CHR$; (f) $CHCHR+H \rightleftarrows CHCH_2R$; (g) $CHCR+H \rightleftarrows CHCHR$; (h) $CHCR+H \rightleftarrows CRCH_2$; (i) $CHRCH_3+H \rightleftarrows CH_3CH_2R$; (j) $CRCH_2+H \rightleftarrows CH_2CHR$; (k) $CRCH_2+H \rightleftarrows CRCH_3$; (l) $CRCH_3+H \rightleftarrows CHRCH_3$.

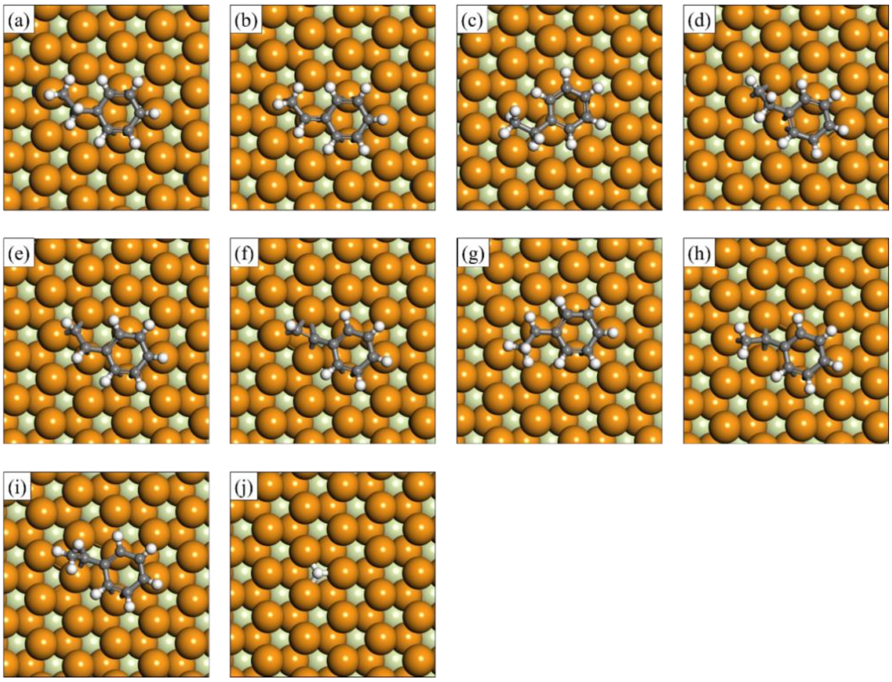

Supplementary Fig. 29 | Top views of all the optimized geometries of intermediates on Pd/Nb_2C for the hydrogenation of phenylacetylene. (a) CH_2CH_2R; (b) CH_2CHR; (c) CH_3CH_2R; (d) $CHCH_2R$; (e) CHCHR; (f) CHCR; (g) $CHRCH_3$; (h) $CRCH_2$; (i) $CRCH_3$; (j) H.

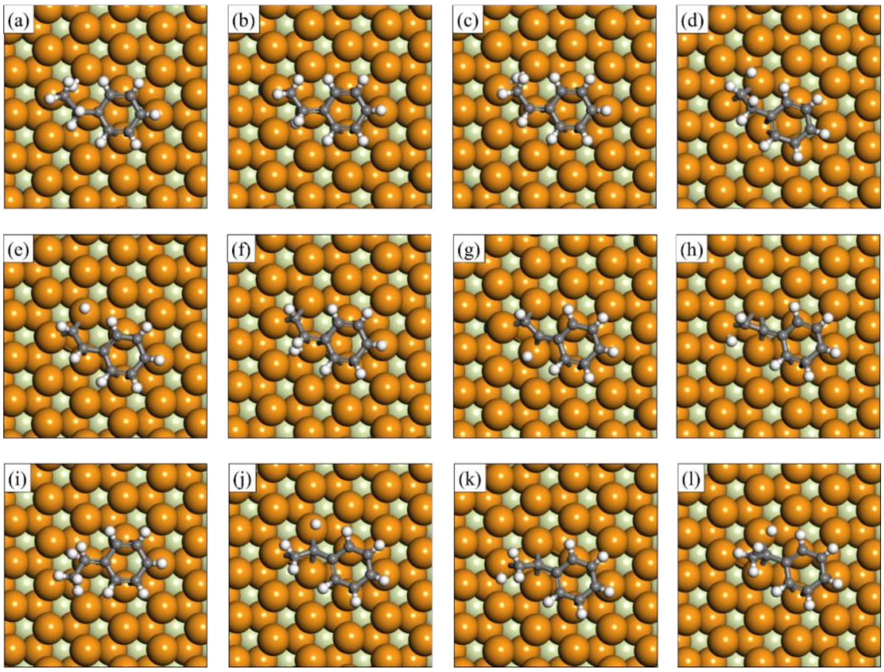

Supplementary Fig. 30 | Top views of calculated transition state geometries on Pd/Nb_2C for the hydrogenation of phenylacetylene. (a) CH_2CH_2R+H ⇄ CH_3CH_2R; (b) CH_2CHR+H ⇄ CH_2CH_2R; (c) CH_2CHR+H ⇄ $CHRCH_3$; (d) $CHCH_2R$+H ⇄ CH_2CH_2R; (e) CHCHR+H ⇄ CH_2CHR; (f) CHCHR+H ⇄ $CHCH_2R$; (g) CHCR+H ⇄ CHCHR; (h) CHCR+H ⇄ $CRCH_2$; (i) $CHRCH_3$+H ⇄ CH_3CH_2R; (j) $CRCH_2$+H ⇄ CH_2CHR; (k) $CRCH_2$+H ⇄ $CRCH_3$; (l) $CRCH_3$+H ⇄ $CHRCH_3$.

Intermediate under CHCR Environment on Pd (111)

1CHCR+Intermediate

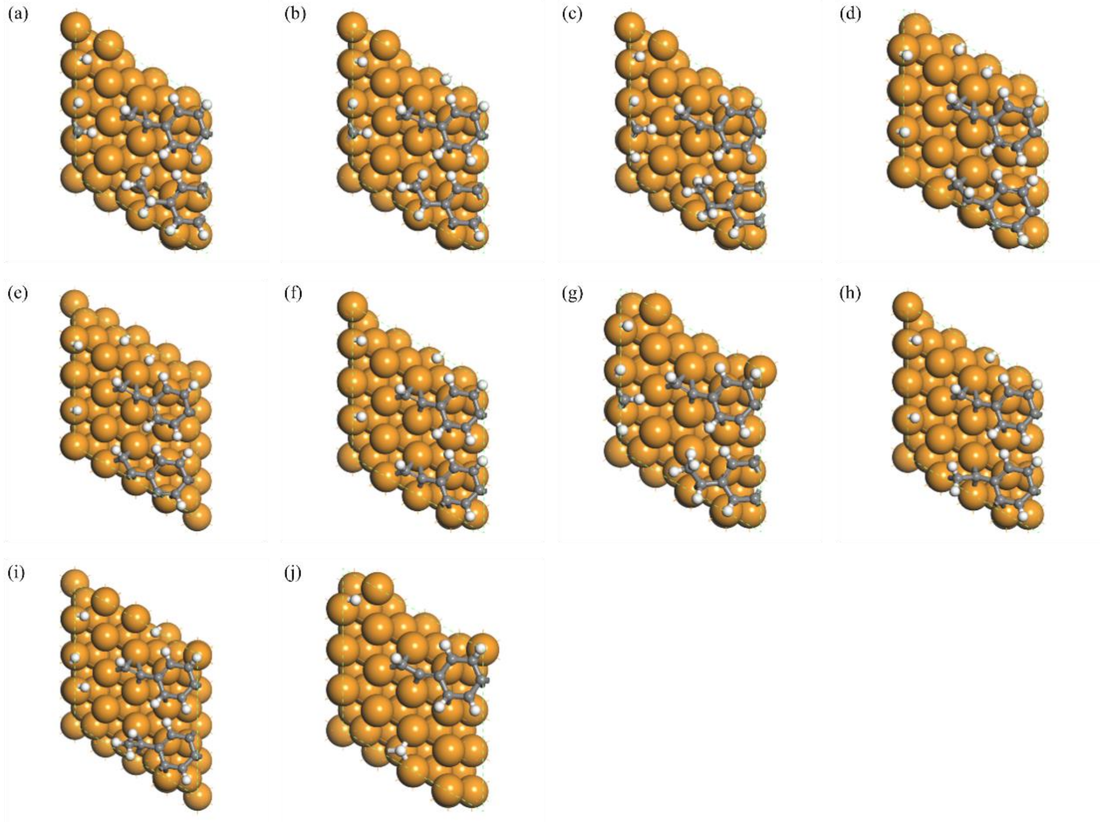

Supplementary Fig. 31 | Top views of all the optimized geometries of intermediates in the presence of 1CHCR on Pd (111) for the hydrogenation of phenylacetylene. (a) CH_2CH_2R; (b) CH_2CHR; (c) CH_3CH_2R; (d) $CHCH_2R$; (e) CHCHR; (f) CHCR; (g) $CHRCH_3$; (h) $CRCH_2$; (i) $CRCH_3$; (j) H.

2CHCR+Intermediates

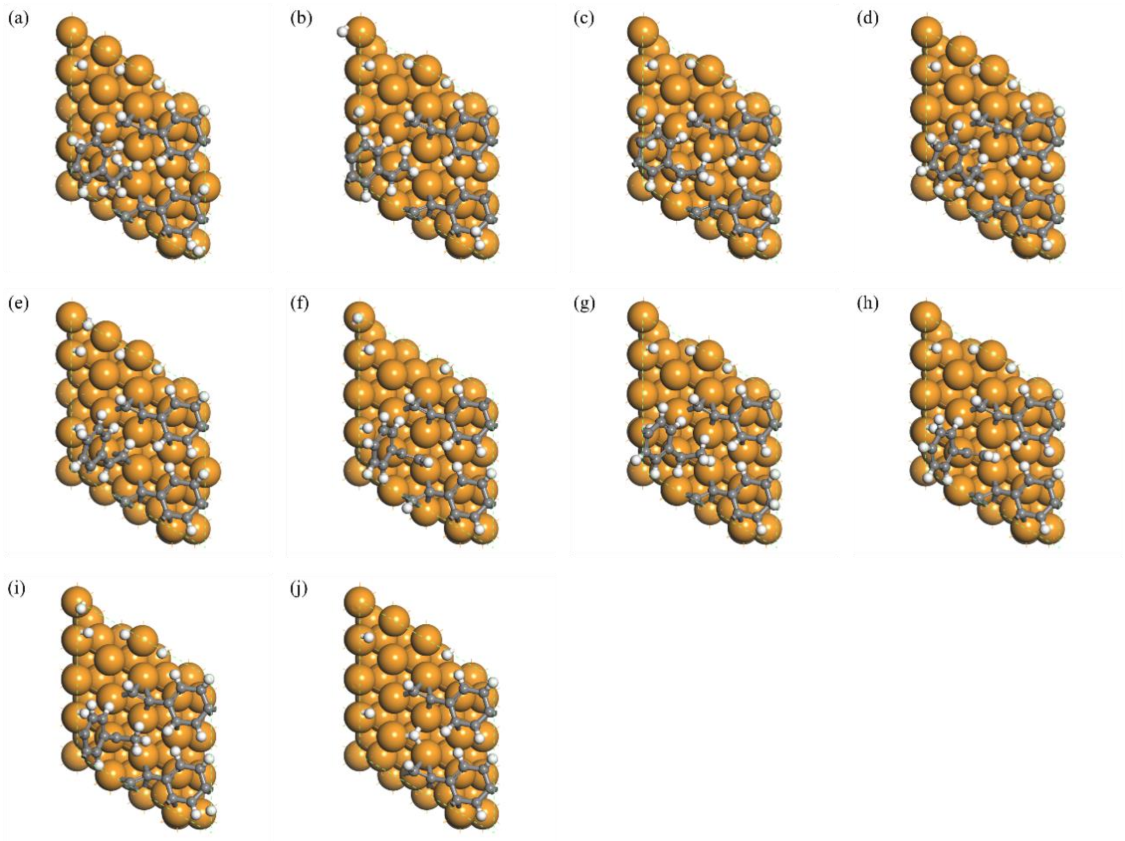

Supplementary Fig. 32 | Top views of all the optimized geometries of intermediates in the presence of 2CHCR on Pd (111) for the hydrogenation of phenylacetylene. (a) CH_2CH_2R; (b) CH_2CHR; (c) CH_3CH_2R; (d) $CHCH_2R$; (e) CHCHR; (f) CHCR; (g) $CHRCH_3$; (h) $CRCH_2$; (i) $CRCH_3$; (j) H.

Transition States under CHCR Environment on Pd (111)

1CHCR+Transition States

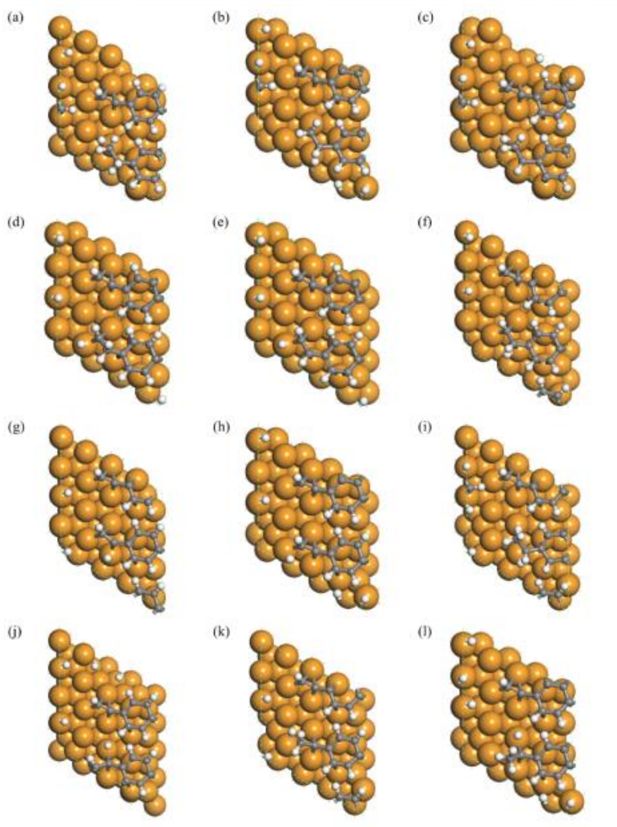

Supplementary Fig. 33 | Top views of the calculated transition state geometries on Pd (111) in the presence of 1CHCR for the hydrogenation of phenylacetylene. (a) CH_2CH_2R+H $\rightleftarrows$ CH_3CH_2R; (b) CH_2CHR+H $\rightleftarrows$ CH_2CH_2R; (c) CH_2CHR+H $\rightleftarrows$ $CHRCH_3$; (d) $CHCH_2R$+H $\rightleftarrows$ CH_2CH_2R; (e) CHCHR+H $\rightleftarrows$ CH_2CHR; (f) CHCHR+H $\rightleftarrows$ $CHCH_2R$; (g) CHCR+H $\rightleftarrows$ CHCHR; (h) CHCR+H $\rightleftarrows$ $CRCH_2$; (i) $CHRCH_3$+H $\rightleftarrows$ CH_3CH_2R; (j) $CRCH_2$+H $\rightleftarrows$ CH_2CHR; (k) $CRCH_2$+H $\rightleftarrows$ $CRCH_3$; (l) $CRCH_3$+H $\rightleftarrows$ $CHRCH_3$.

2CHCR+Transition States

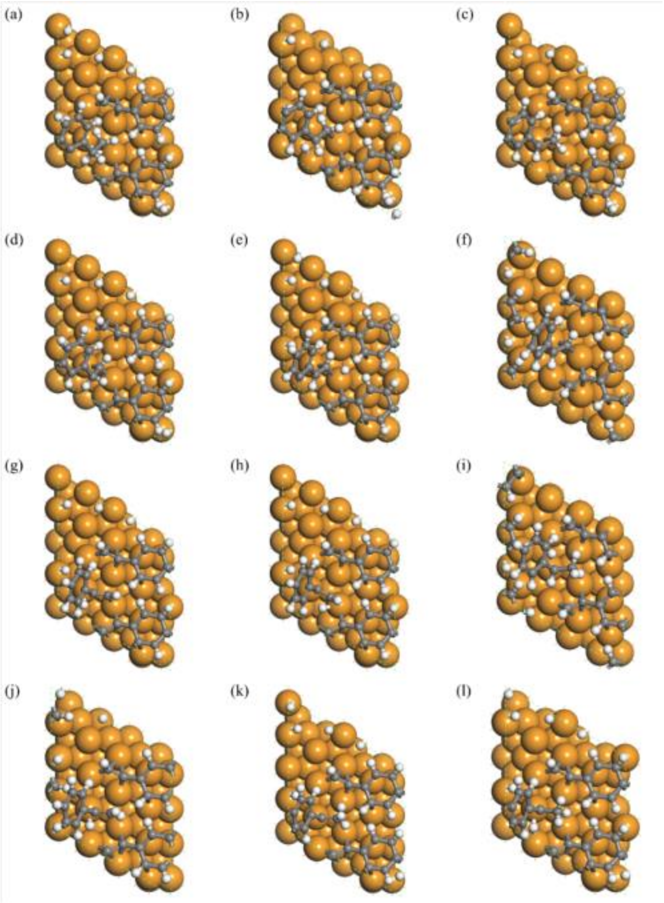

Supplementary Fig. 34 | Top views of the calculated transition state geometries on Pd (111) in the presence of 2CHCR for the hydrogenation of phenylacetylene. (a) CH_2CH_2R+H $\rightleftarrows$ CH_3CH_2R; (b) CH_2CHR+H $\rightleftarrows$ CH_2CH_2R; (c) CH_2CHR+H $\rightleftarrows$ $CHRCH_3$; (d) $CHCH_2R$+H $\rightleftarrows$ CH_2CH_2R; (e) CHCHR+H $\rightleftarrows$ CH_2CHR; (f) CHCHR+H $\rightleftarrows$ $CHCH_2R$; (g) CHCR+H $\rightleftarrows$ CHCHR; (h) CHCR+H $\rightleftarrows$ $CRCH_2$; (i) $CHRCH_3$+H $\rightleftarrows$ CH_3CH_2R; (j) $CRCH_2$+H $\rightleftarrows$ CH_2CHR; (k) $CRCH_2$+H $\rightleftarrows$ $CRCH_3$; (l) $CRCH_3$+H $\rightleftarrows$ $CHRCH_3$.

Intermediate under CHCR Environment on Pd/Nb_2C

1CHCR+Intermediate

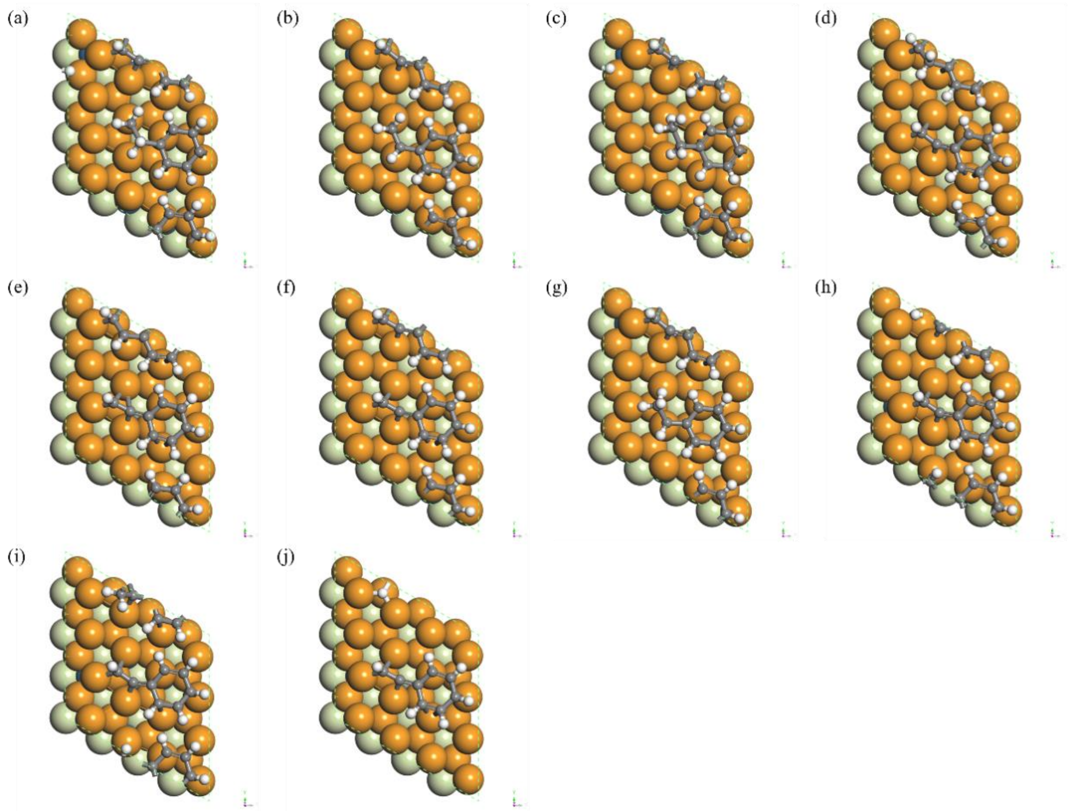

Supplementary Fig. 35 | Top views of all the optimized geometries of intermediates in the presence of 1CHCR on Pd/Nb_2C for the hydrogenation of phenylacetylene. (a) CH_2CH_2R; (b) CH_2CHR; (c) CH_3CH_2R; (d) $CHCH_2R$; (e) CHCHR; (f) CHCR; (g) $CHRCH_3$; (h) $CRCH_2$; (i) $CRCH_3$; (j) H.

2CHCR+Intermediates

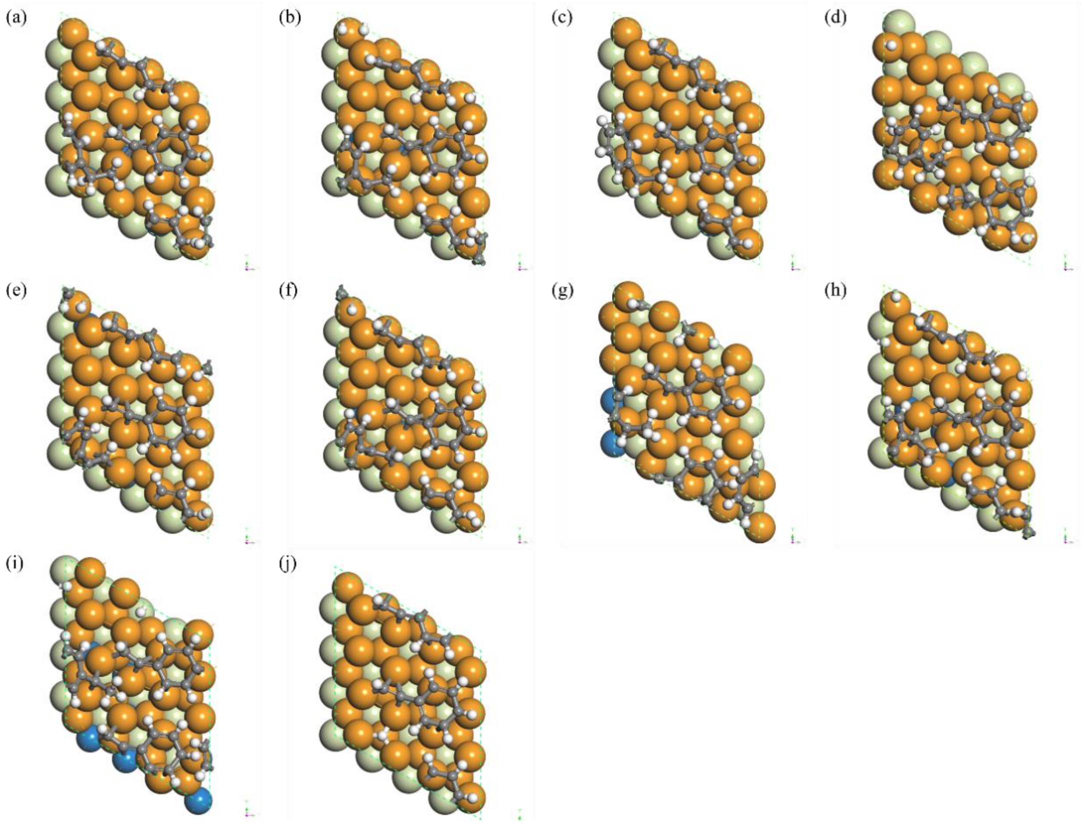

Supplementary Fig. 36 | Top views of all the optimized geometries of intermediates in the presence of 2CHCR on Pd/Nb_2C for the hydrogenation of phenylacetylene. (a) CH_2CH_2R; (b) CH_2CHR; (c) CH_3CH_2R; (d) $CHCH_2R$; (e) CHCHR; (f) CHCR; (g) $CHRCH_3$; (h) $CRCH_2$; (i) $CRCH_3$; (j) H.

Transition States under CHCR Environment on Pd/Nb_2C

1CHCR+Transition States

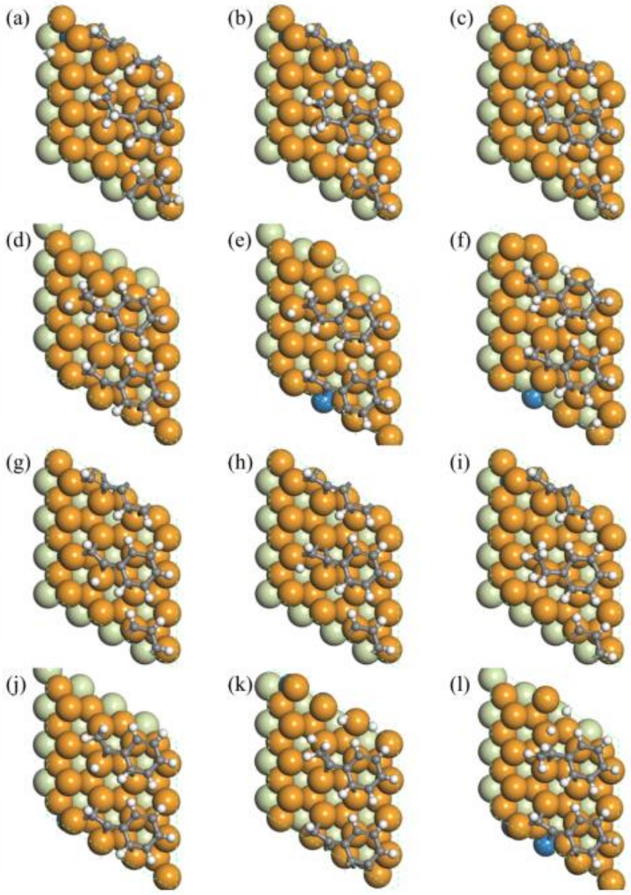

Supplementary Fig. 37 | Top views of the calculated transition state geometries on Pd/Nb_2C in the presence of 1CHCR for the hydrogenation of phenylacetylene. (a) CH_2CH_2R+H ⇄ CH_3CH_2R; (b) CH_2CHR+H ⇄ CH_2CH_2R; (c) CH_2CHR+H ⇄ $CHRCH_3$; (d) $CHCH_2R$+H ⇄ CH_2CH_2R; (e) CHCHR+H ⇄ CH_2CHR; (f) CHCHR+H ⇄ $CHCH_2R$; (g) CHCR+H ⇄ CHCHR; (h) CHCR+H ⇄ $CRCH_2$; (i) $CHRCH_3$+H ⇄ CH_3CH_2R; (j) $CRCH_2$+H ⇄ CH_2CHR; (k) $CRCH_2$+H ⇄ $CRCH_3$; (l) $CRCH_3$+H ⇄ $CHRCH_3$.

2CHCR+Transition States

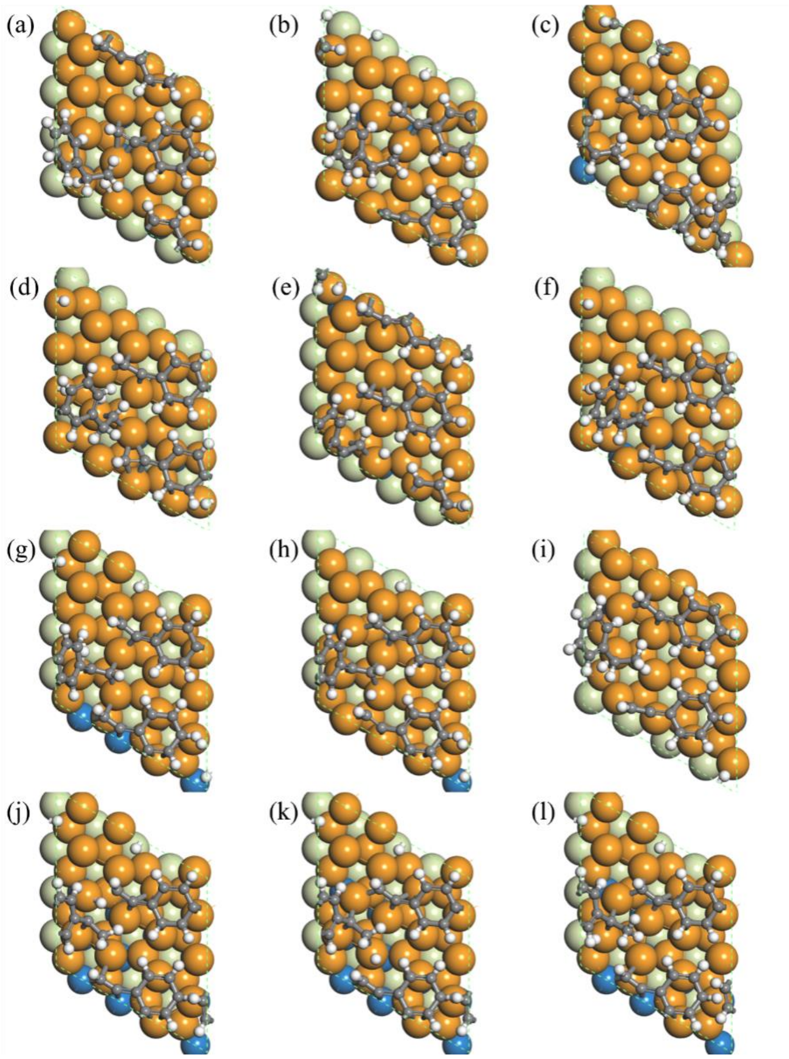

Supplementary Fig. 38 | Top views of the calculated transition state geometries on Pd/Nb_2C in the presence of 2CHCR for the hydrogenation of phenylacetylene. (a) CH_2CH_2R+H ⇄ CH_3CH_2R; (b) CH_2CHR+H ⇄ CH_2CH_2R; (c) CH_2CHR+H ⇄ $CHRCH_3$; (d) $CHCH_2R$+H ⇄ CH_2CH_2R; (e) CHCHR+H ⇄ CH_2CHR; (f) CHCHR+H ⇄ $CHCH_2R$; (g) CHCR+H ⇄ CHCHR; (h) CHCR+H ⇄ $CRCH_2$; (i) $CHRCH_3$+H ⇄ CH_3CH_2R; (j) $CRCH_2$+H ⇄ CH_2CHR; (k) $CRCH_2$+H ⇄ $CRCH_3$; (l) $CRCH_3$+H ⇄ $CHRCH_3$.

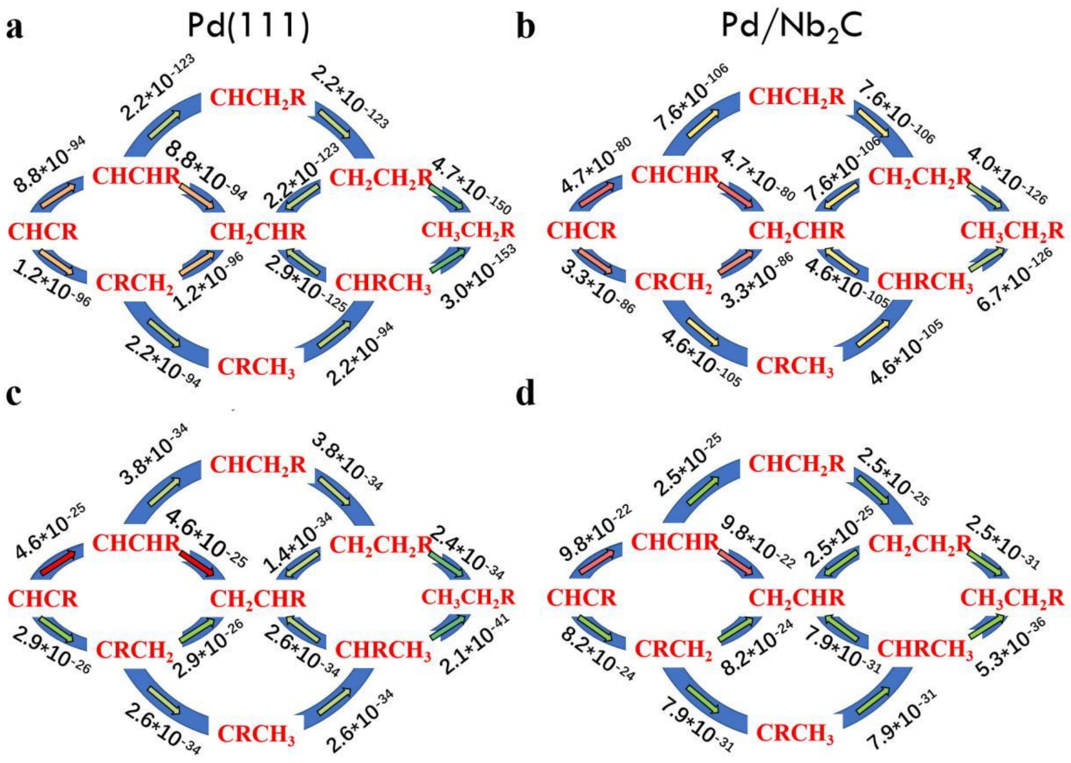

Supplementary Fig. 39 | (a) Non-coverage-dependent reaction pathways for the hydrogenation of phenylacetylene on Pd (111). (b) Non-coverage-dependent reaction pathways for the hydrogenation of phenylacetylene on Pd/Nb_2C. (c) Coverage-dependent reaction pathways for the hydrogenation of phenylacetylene on Pd (111). (d) Coverage-dependent reaction pathways for the hydrogenation of phenylacetylene on Pd/Nb_2C. Red arrows represent the important pathways according to the results of the microkinetic model. The values represent the reaction rates for each elementary step and the unit is s^{-1}. (T = 298.15 K)

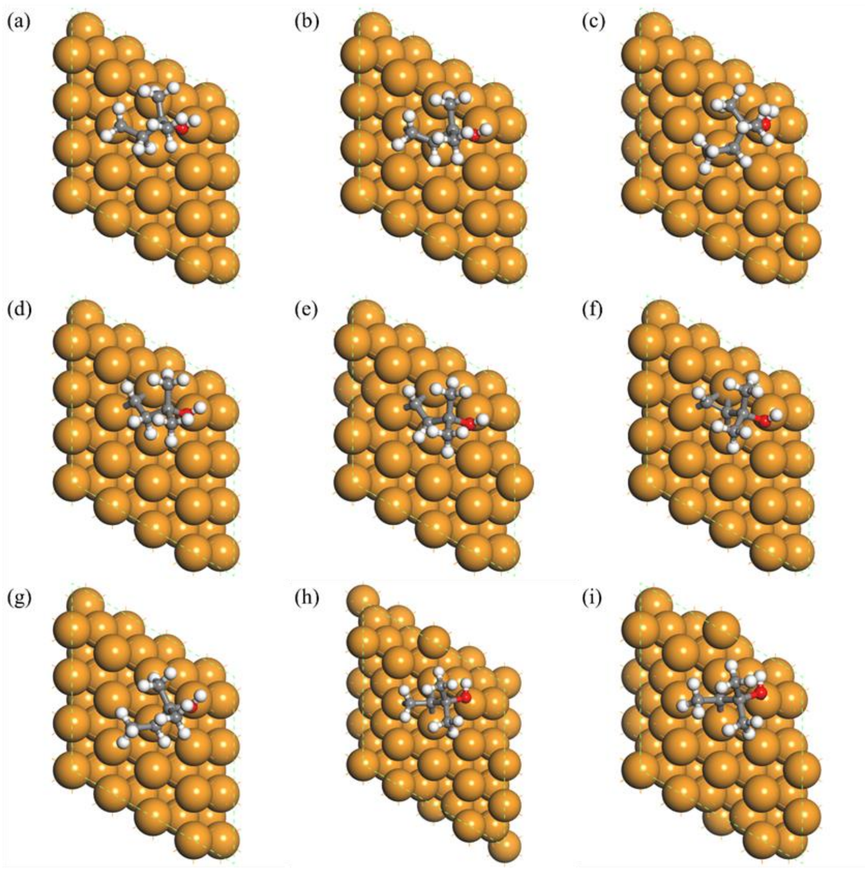

Supplementary Fig. 40 | Top views of all the optimized geometries of intermediates on Pd (111) for the hydrogenation of 2-methyl-3-butyn-2-ol (MBY). (a) CH_2CH_2R; (b) CH_2CHR; (c) CH_3CH_2R; (d) $CHCH_2R$; (e) CHCHR; (f) CHCR; (g) $CHRCH_3$; (h) $CRCH_2$; (i) $CRCH_3$.

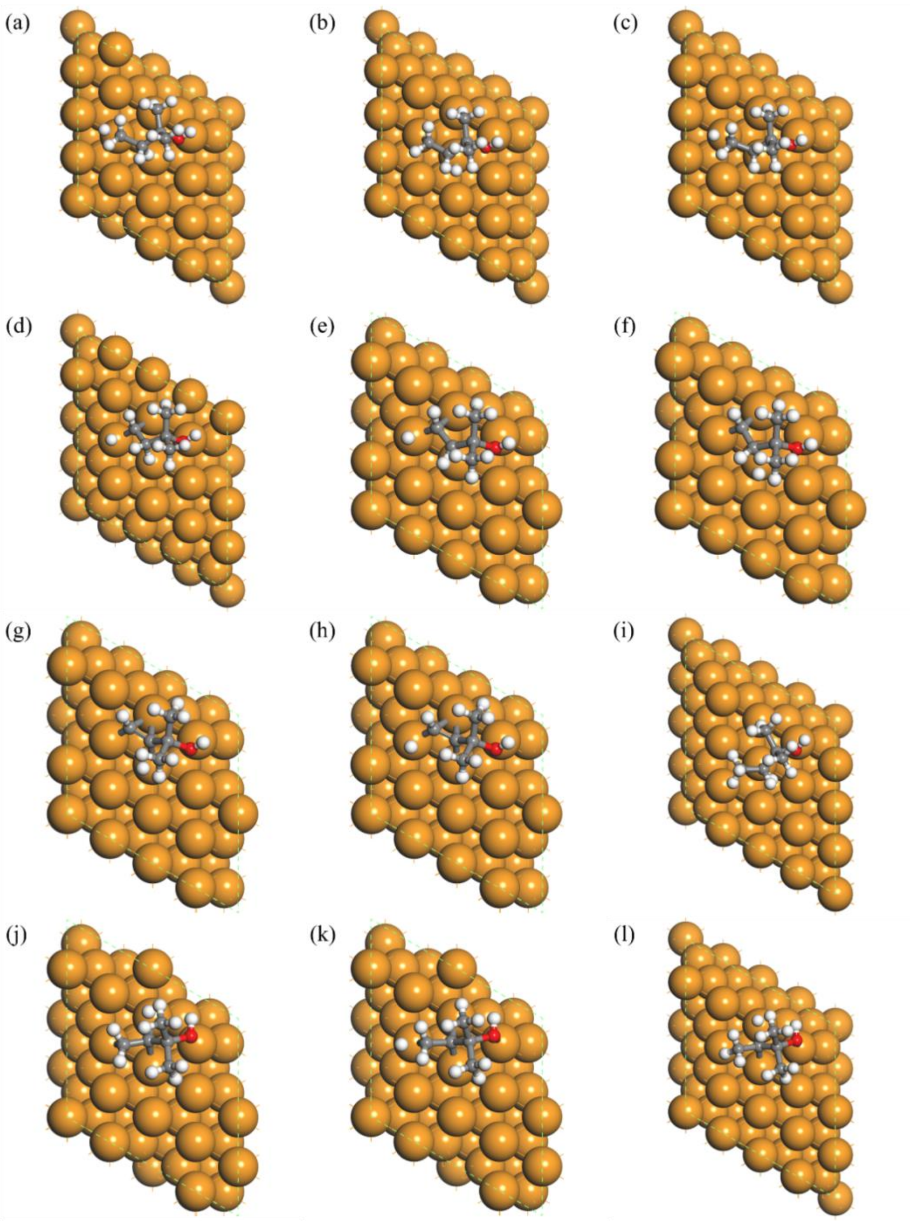

Supplementary Fig. 41 | Top views of calculated transition state geometries on Pd (111) for the hydrogenation of MBY. (a) CH_2CH_2R+H $\rightleftarrows$ CH_3CH_2R; (b) CH_2CHR+H $\rightleftarrows$ CH_2CH_2R; (c) CH_2CHR+H $\rightleftarrows$ $CHRCH_3$; (d) $CHCH_2R$+H $\rightleftarrows$ CH_2CH_2R; (e) CHCHR+H $\rightleftarrows$ CH_2CHR; (f) CHCHR+H $\rightleftarrows$ $CHCH_2R$; (g) CHCR+H $\rightleftarrows$ CHCHR; (h) CHCR+H $\rightleftarrows$ $CRCH_2$; (i) $CHRCH_3$+H $\rightleftarrows$ CH_3CH_2R; (j) $CRCH_2$+H $\rightleftarrows$ CH_2CHR; (k) $CRCH_2$+H $\rightleftarrows$ $CRCH_3$; (l) $CRCH_3$+H $\rightleftarrows$ $CHRCH_3$.

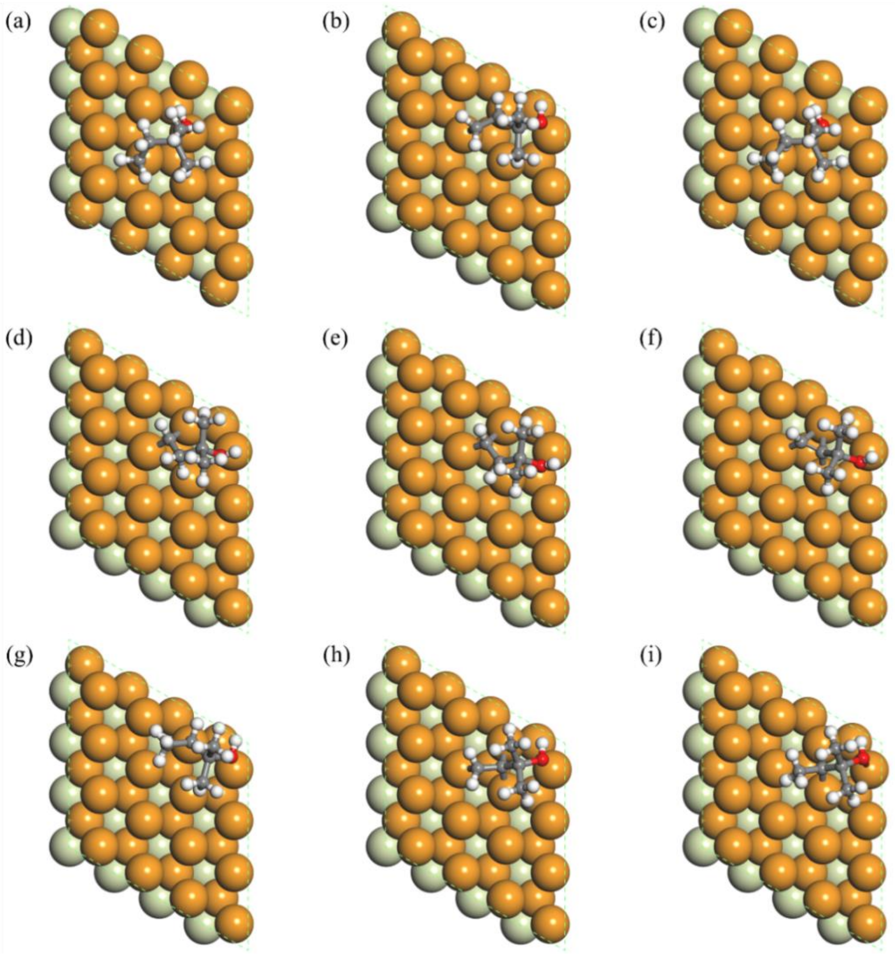

Supplementary Fig. 42 | Top views of all the optimized geometries of intermediates on Pd/Nb_2C for the hydrogenation of MBY. (a) CH_2CH_2R; (b) CH_2CHR; (c) CH_3CH_2R; (d) $CHCH_2R$; (e) CHCHR; (f) CHCR; (g) $CHRCH_3$; (h) $CRCH_2$; (i) $CRCH_3$.

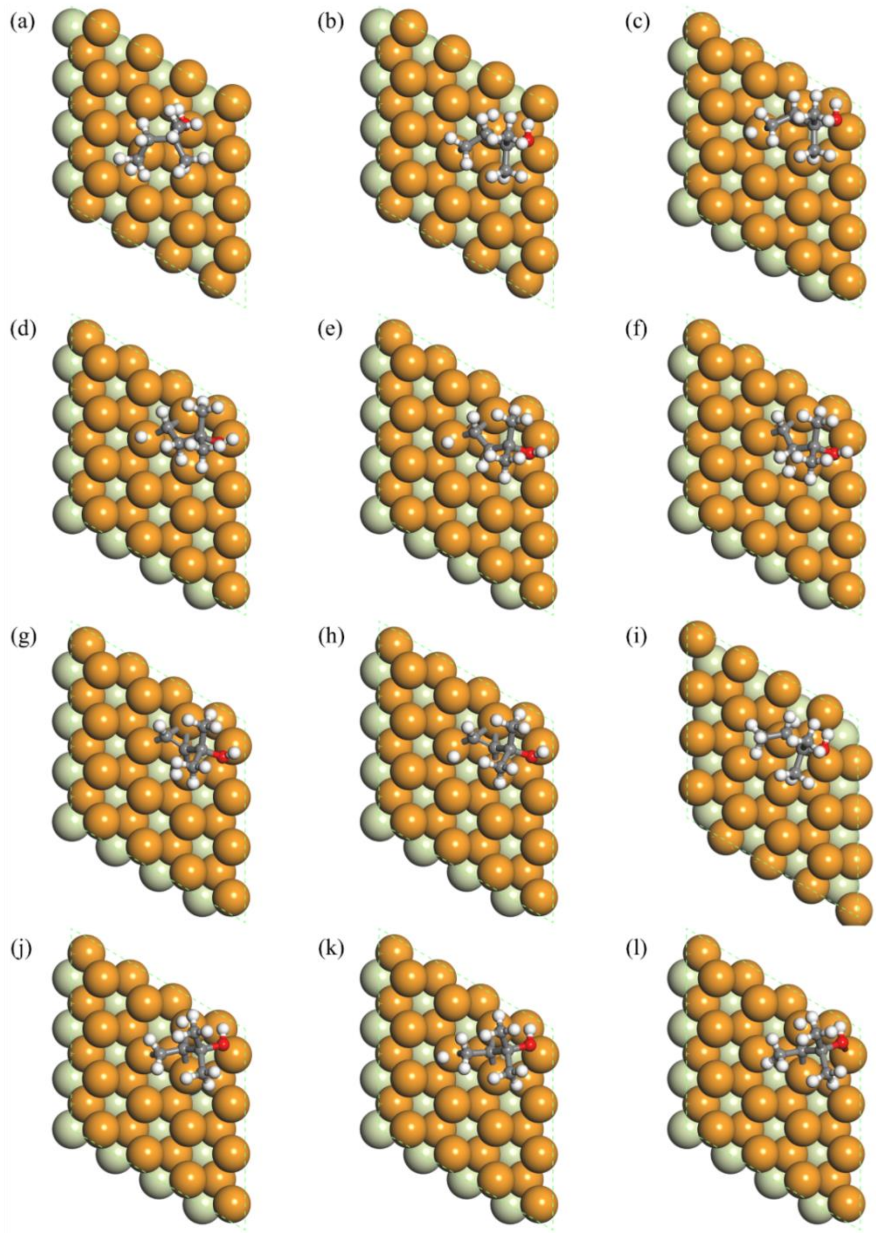

Supplementary Fig. 43 | Top views of calculated transition state geometries on Pd/Nb_2C for the hydrogenation of MBY. (a) CH_2CH_2R+H ⇄ CH_3CH_2R; (b) CH_2CHR+H ⇄ CH_2CH_2R; (c) CH_2CHR+H ⇄ $CHRCH_3$; (d) $CHCH_2R$+H ⇄ CH_2CH_2R; (e) CHCHR+H ⇄ CH_2CHR; (f) CHCHR+H ⇄ $CHCH_2R$; (g) CHCR+H ⇄ CHCHR; (h) CHCR+H ⇄ $CRCH_2$; (i) $CHRCH_3$+H ⇄ CH_3CH_2R; (j) $CRCH_2$+H ⇄ CH_2CHR; (k) $CRCH_2$+H ⇄ $CRCH_3$; (l) $CRCH_3$+H ⇄ $CHRCH_3$.

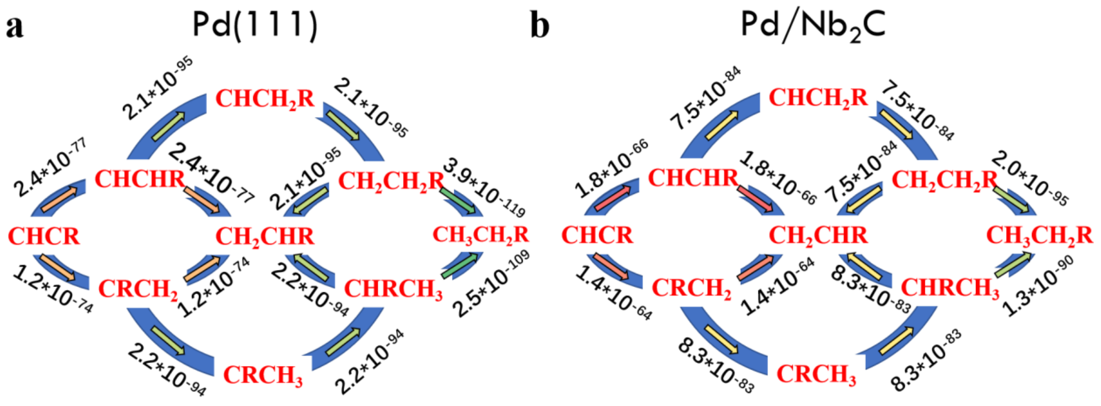

Supplementary Fig. 44 | (a) Reaction pathways for the hydrogenation of MBY on Pd (111). (b) Reaction pathways for the hydrogenation of MBY on Pd/Nb_2C. Red arrows represent the important pathways according to the results of the microkinetic model. The values represent the reaction rates for each elementary step and the unit is s^{-1}. (T = 298.15 K)

GC-MS characterization of some supplemental substrates

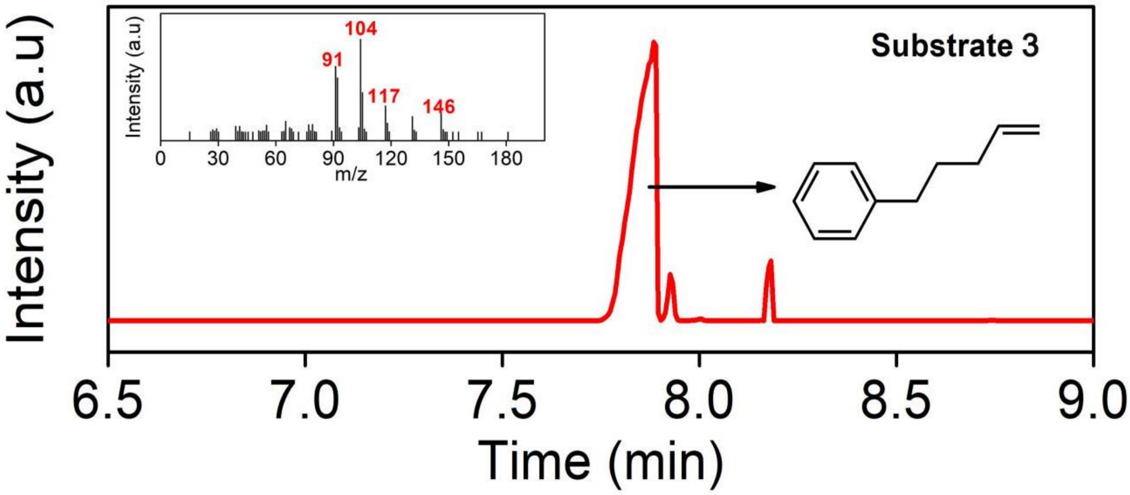

Supplementary Fig. 45 | GC-MS of entries 3

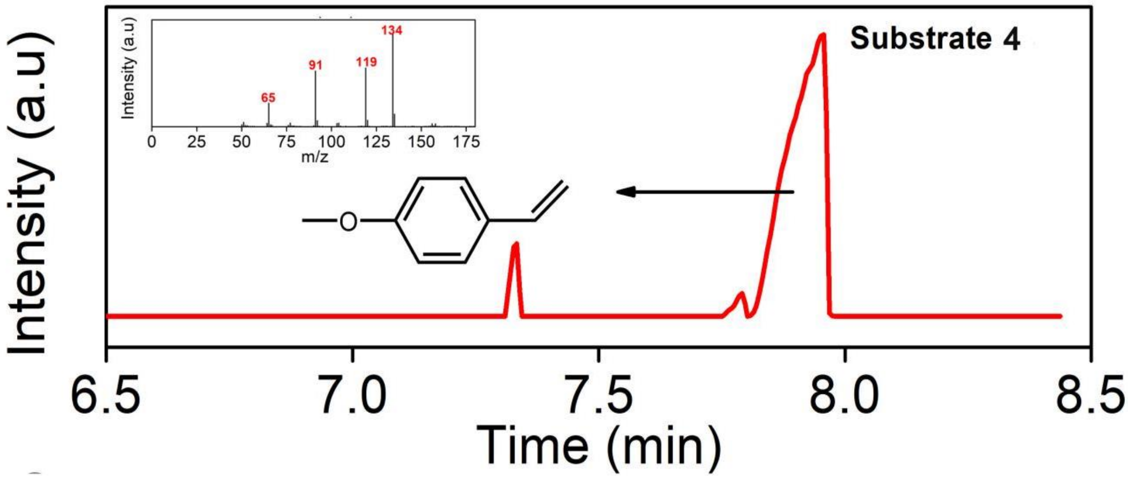

Supplementary Fig. 46 | GC-MS of entries 4

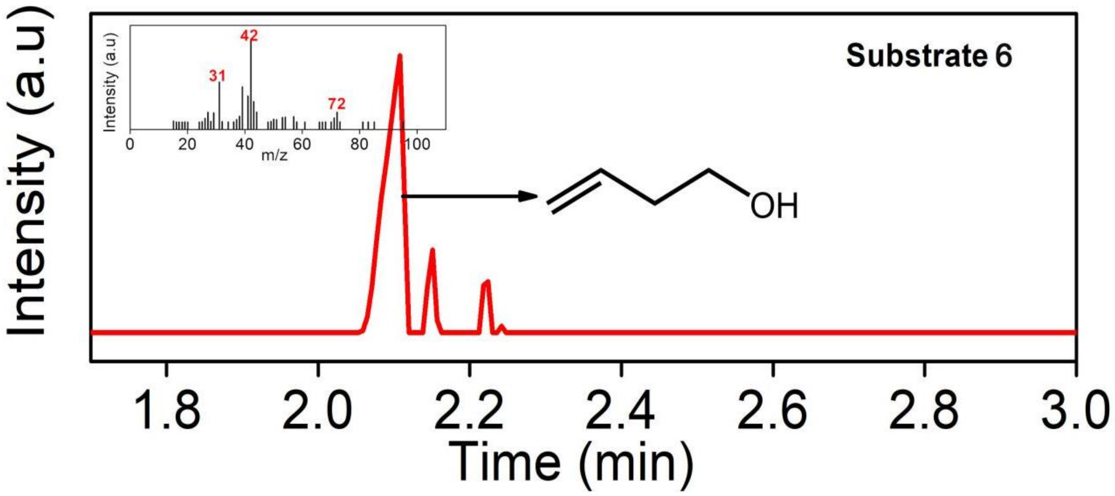

Supplementary Fig. 47 | GC-MS of entries 6

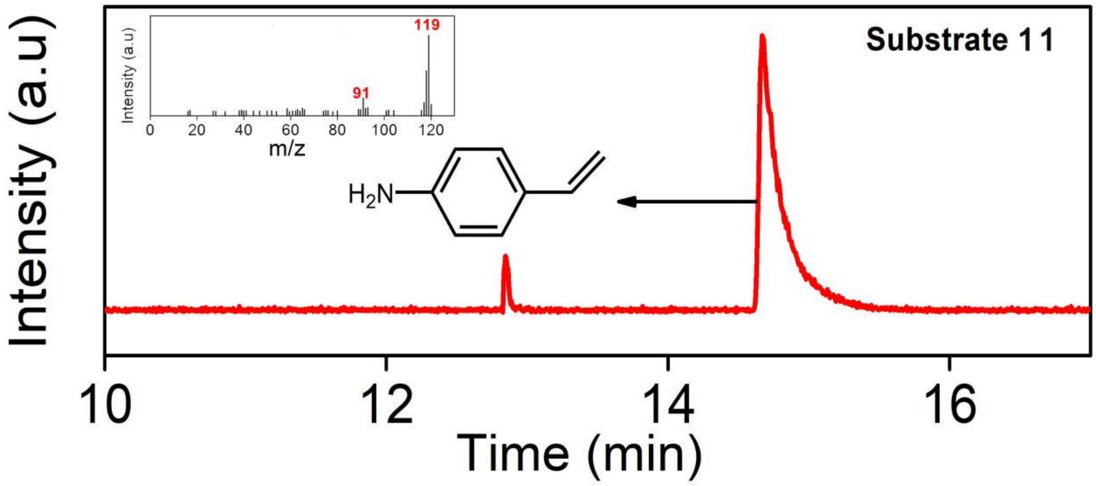

Supplementary Fig. 48 | GC-MS of entries 11

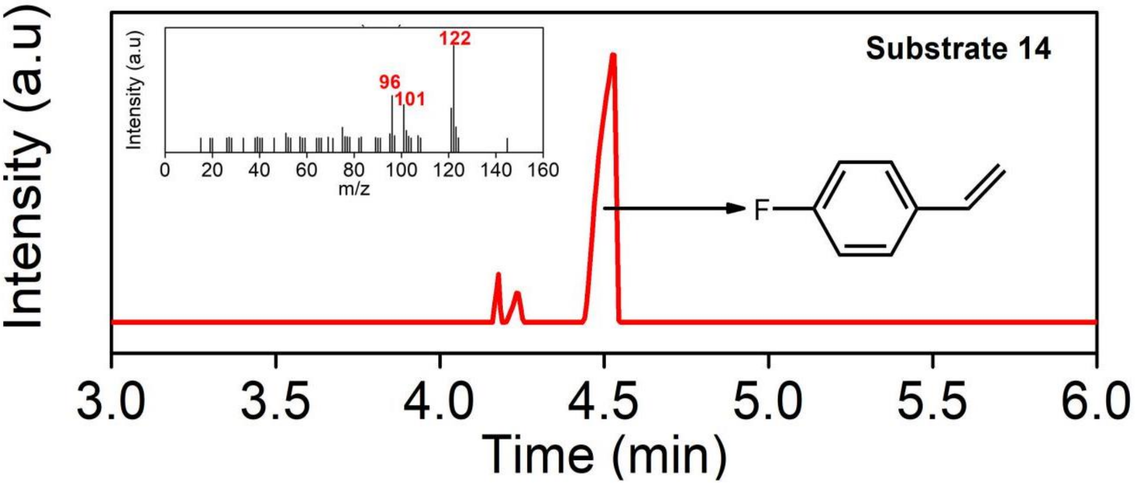

Supplementary Fig. 49 | GC-MS of entries 14

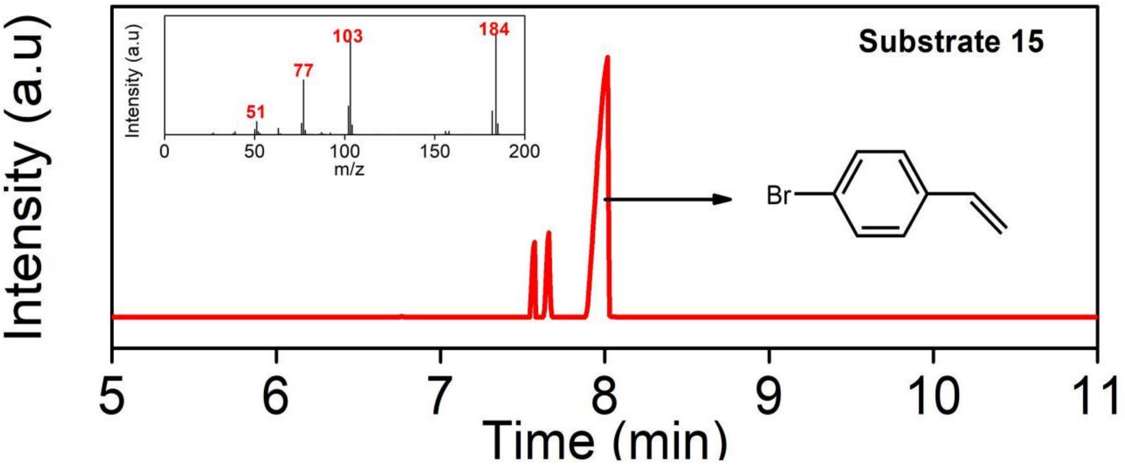

Supplementary Fig. 50 | GC-MS of entries 15

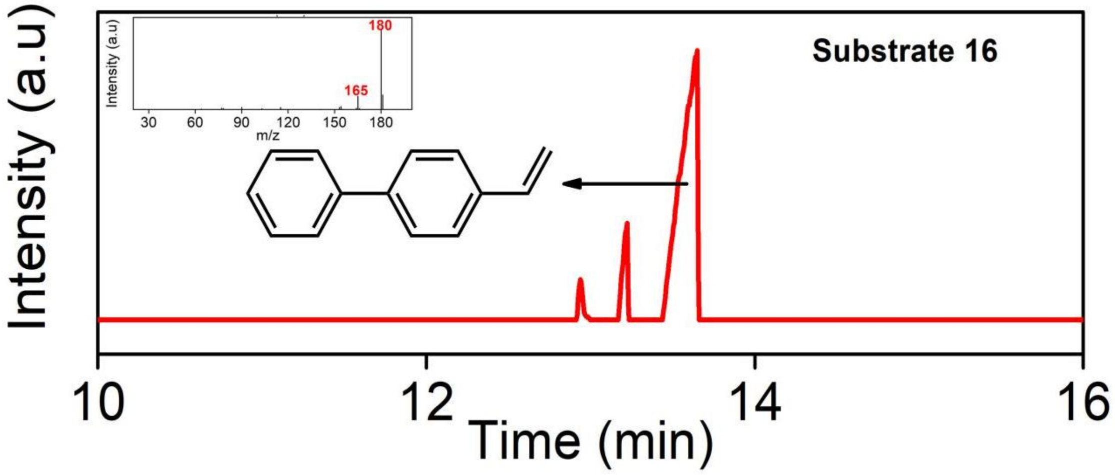

Supplementary Fig. 51 | GC-MS of entries 16

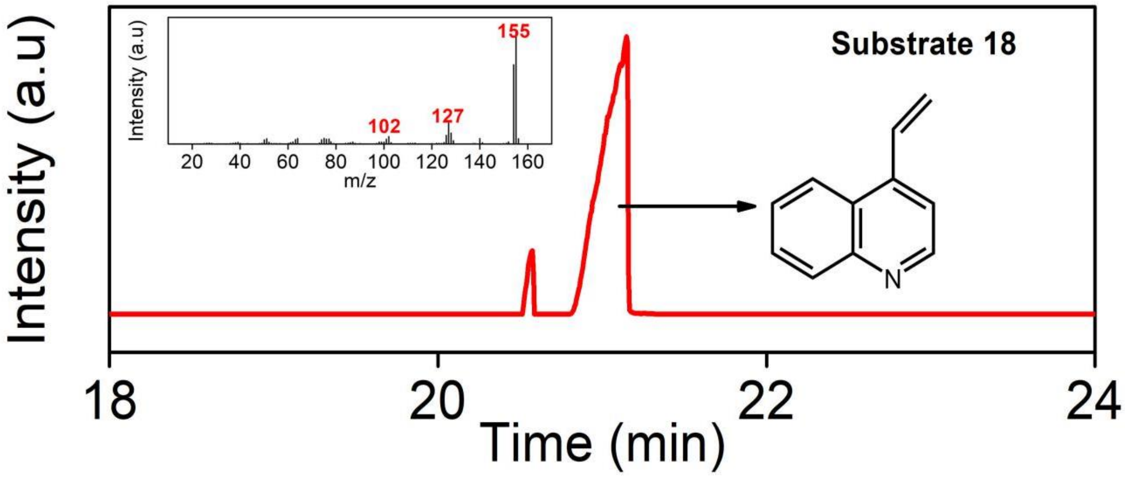

Supplementary Fig. 52 | GC-MS of entries 18

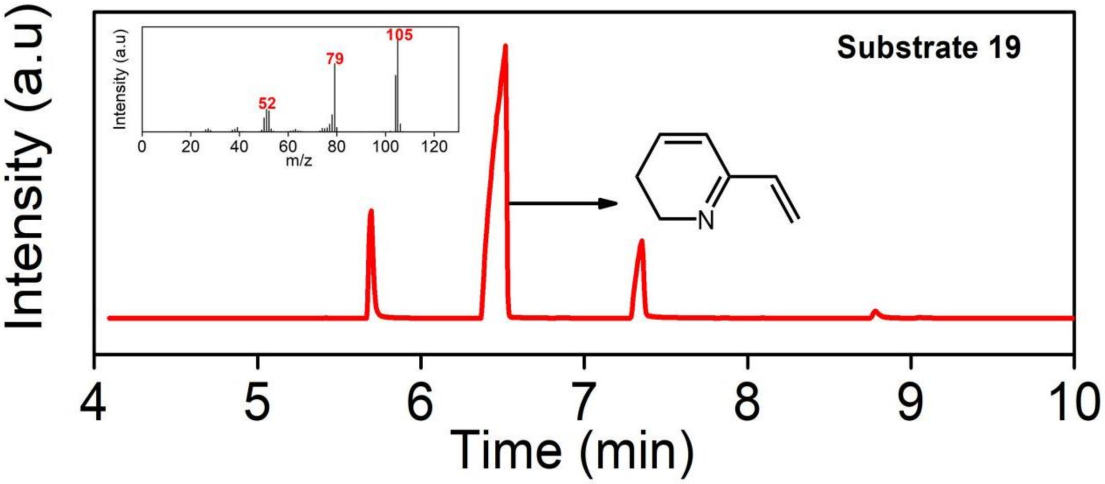

Supplementary Fig. 53 | GC-MS of entries 19

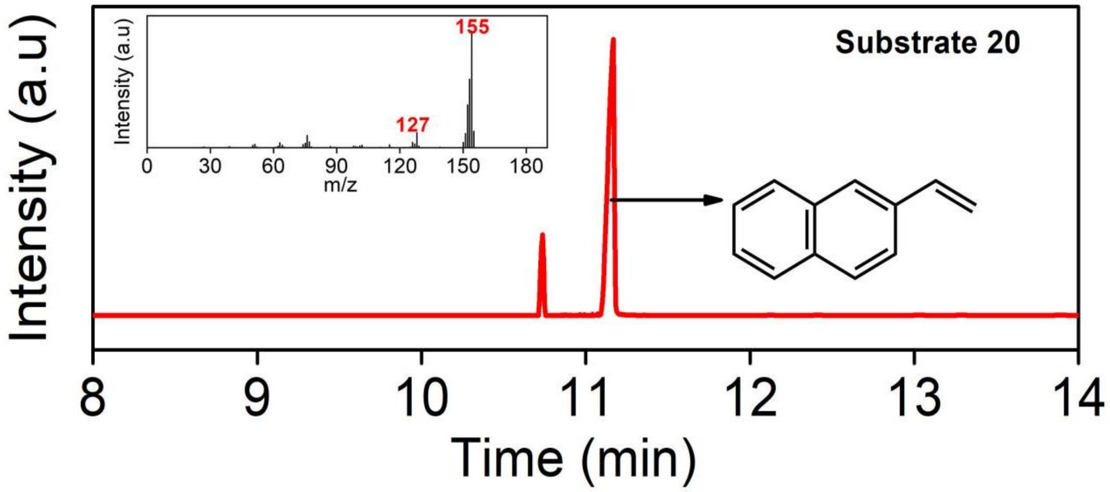

Supplementary Fig. 54 | GC-MS of entries 20

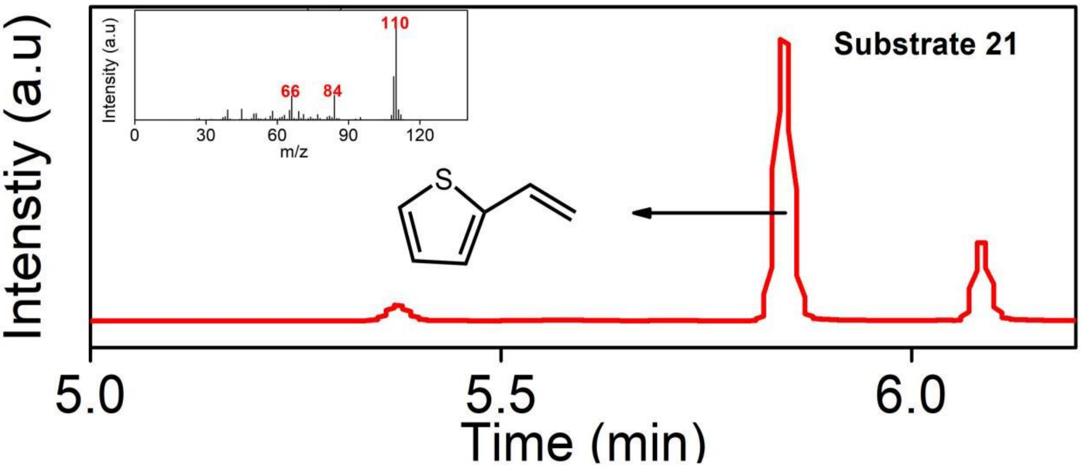

Supplementary Fig. 55 | GC-MS of entries 21

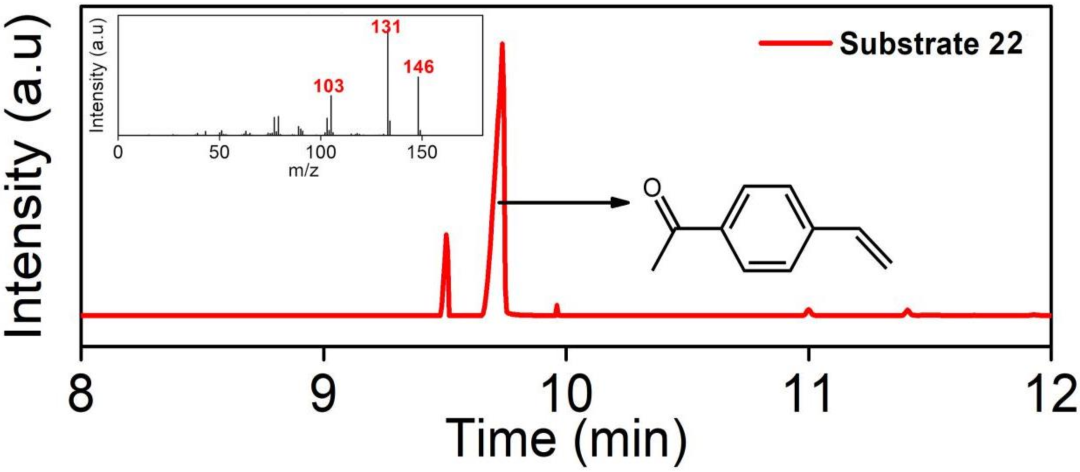

Supplementary Fig. 56 | GC-MS of entries 22

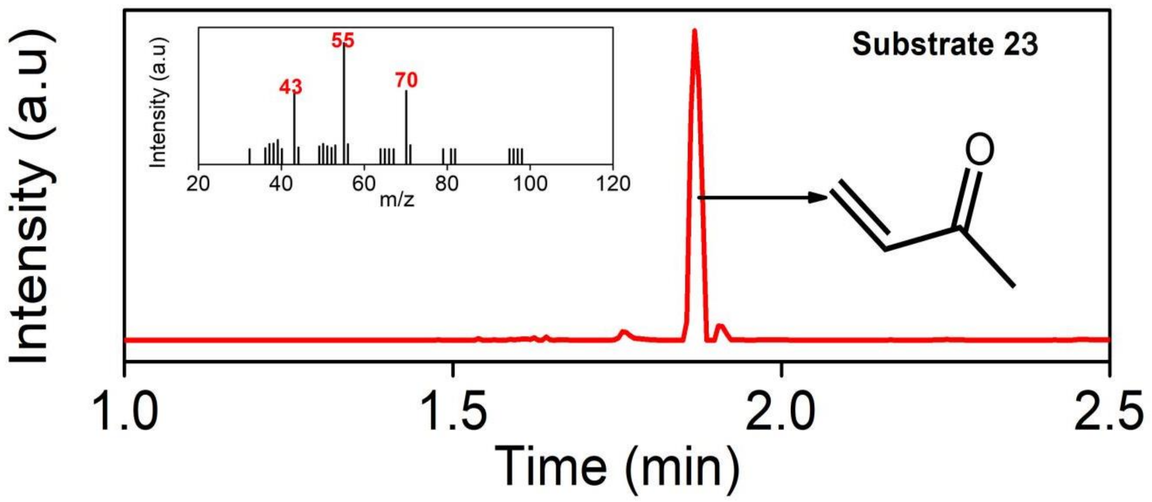

Supplementary Fig. 57 | GC-MS of entries 23

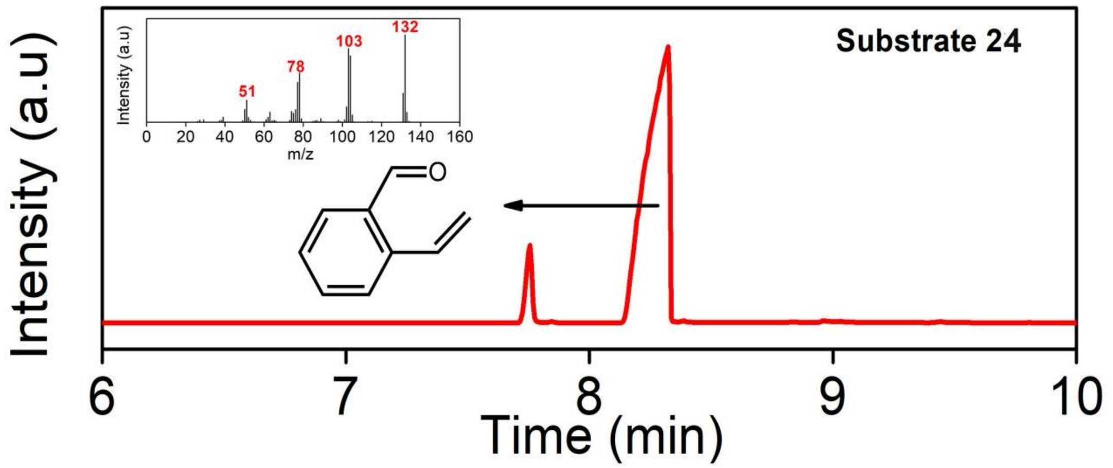

Supplementary Fig. 58 | GC-MS of entries 24

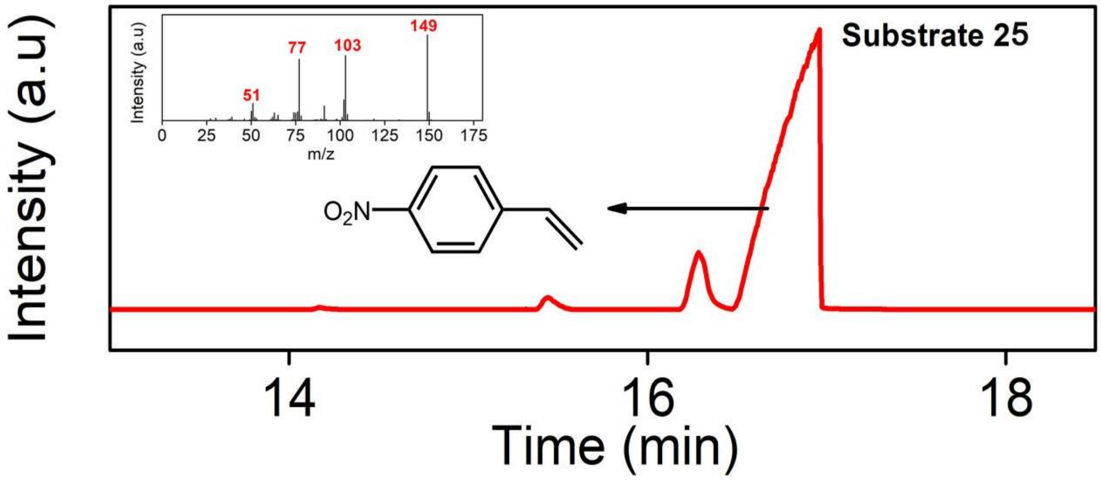

Supplementary Fig. 59 | GC-MS of entries 25

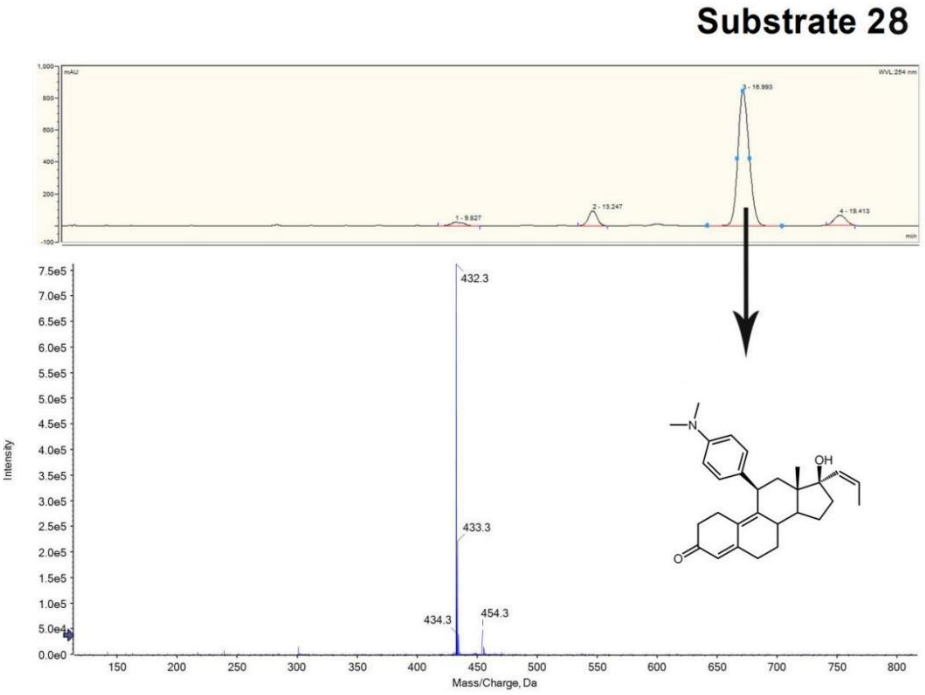

Supplementary Fig. 60 | LC-MS of entries 28

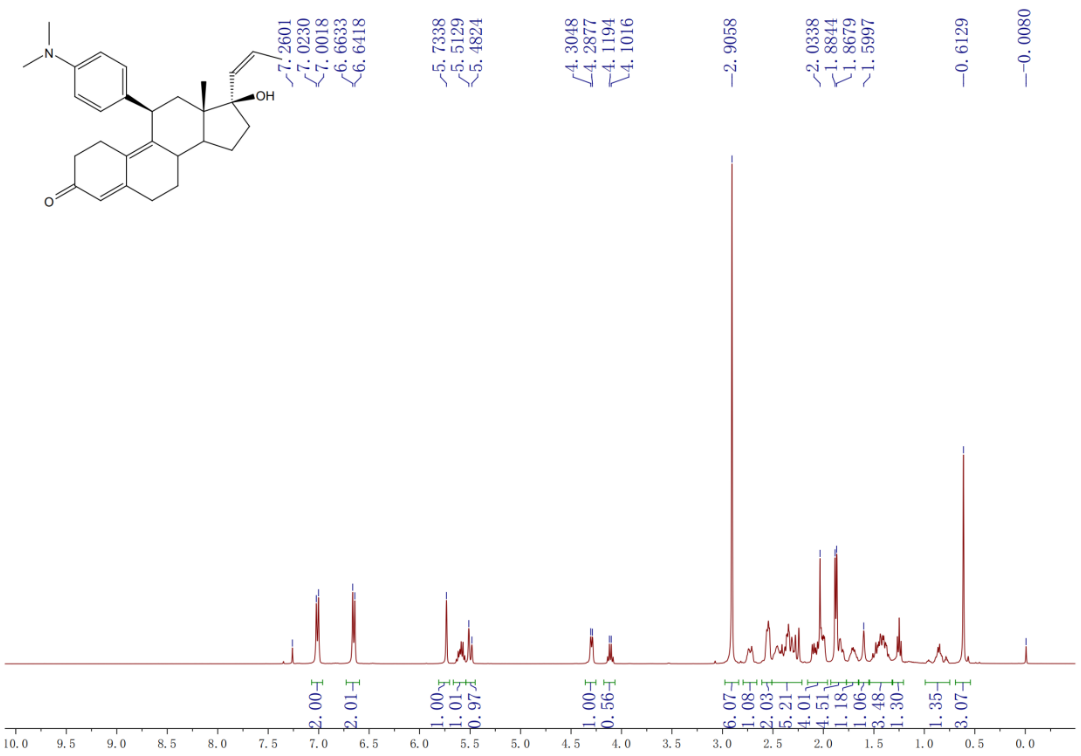

Supplementary Fig. 61. 1H-NMR data for hydrogenation products of 28.

1H NMR (400 MHz, $CDCl_3$) chemical shift δ 7.02 (d, 2H), 6.66 (d, 2H), 5.73 (s, 1H), 5.51 (m, 1H), 5.48 (d, 1H), 4.30 (d, 1H), 2.91 (s, 6H), 2.78-2.66 (m, 1H), 2.58 (m, 2H), 2.51-2.22 (m, 5H), 2.15-1.96 (m, 2H), 1.88 (4H), 1.82-1.78 (m, 1H), 1.77-1.66 (m, 1H), 1.52-1.28 (m, 3H), 0.94-0.76 (m,1H), 0.61 (s, 3H). Other H at δ 1.26, 2.03 and 4.10 are attributed to residual ethyl acetate during the purification process of the product.

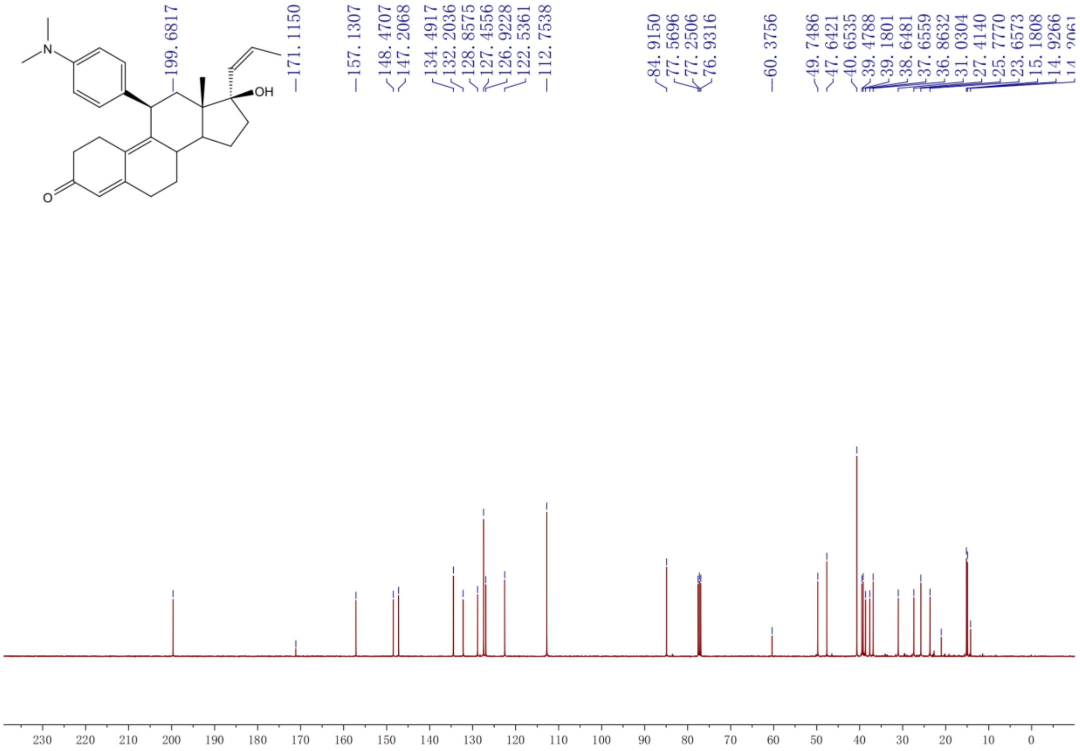

Supplementary Fig. 62. 13C-NMR data for hydrogenation product of 28.

Aglepristone $C_{29}H_{37}NO_2$

13C NMR (101 MHz, $CDCl_3$) chemical shift δ 199.68, 157.13, 148.47, 147.21, 134.49, 132.20, 128.86, 127.46 (2C), 126.92, 122.54, 112.75 (2C), 84.92, 49.75, 47.64, 40.65 (2C), 39.48, 39.18, 38.65, 37.66, 36.86, 31.03, 27.41, 25.78, 23.66, 15.28, 14.93.

Supplementary Table 1. Morse force field parameters for Pd/Nb_2C, Pd/O_2Nb_2C, Pd/Br_2Nb_2C and Pd/Cl_2Nb_2C.

		***D* (eV)**	***α* (1/Å)**	**r_0 (Å)**
Bare surface	Pd-Nb	1.807	1.566	2.30
	Pd-C	0.047	0.960	5.30
O group	Pd-O	2.913	2.642	1.40
	Pd-Nb	0.0187	0.520	4.80
	Pd-C	0.008	2.191	5.60
Br group	Pd-Br	0.500	2.097	2.10
	Pd-Nb	0.0211	0.196	4.70
	Pb-C	0.00247	1.417	7.00
Cl group	Pd-Cl	2.102	2.514	1.60
	Pd-Nb	0.0220	0.450	4.90
	Pd-C	0.00163	2.138	6.70

Supplementary Table 2. Structural parameters of a series of Pd/Nb_2C extracted from the EXAFS fitting of Pd *K*-edge.

Catalyst	Path	CN[a]	R[b](Å)	$\sigma^2\times 10^2$ (Å^2)[c]	ΔE_0 (eV)
Pd/Nb_2C	Pd-O	2.1	2.00	0.001	1.632± 0.764
	Pd-Pd	3	2.74	0.017	
Pd/Br-Nb_2C	Pd-Pd	11.1±1.0	2.75±0.01	0.64±0.5	-0.1±0.8
Pd/O-Nb_2C	Pd-Pd	10.4±0.7	2.76±0.01	0.78±0.4	1.3±0.

[a]CN is the coordination number; [b]R is interatomic distance; [c]σ^2 is Debye-Waller factor (a measure of thermal and static disorder in absorber-scatter distances); S_0^2 was obtained from Nb metal and fixed as 0.80; ΔE_0 shift in absorption edge energy, Data ranges $3.000 \leq k \leq 12.651$ Å^{-1}, $1.25 \leq R \leq 2.70$ Å. The number of variable parameters is 3, out of a total of 8.8125 independent data points, R factor for these fits are 1.79%, 0.4%, 0.6%.

Supplementary Table 3. Structural parameters of a series of Pd/Nb_2C extracted from the EXAFS fitting of Nb *K*-edge.

Catalyst	Path	CN[a]	R[b](Å)	$\sigma^2\times 10^{-2}$ (Å^2)[c]	ΔE_0 (eV)
Pd/Nb_2C	Nb-C	2.1	2.11	1.1	13.3
	Nb-Pd	1.8	2.72	1.1	
	Nb-C-Nb	0.5	3.11	0.6	
Pd/O-Nb_2C	Nb-C	2.1	2.11	0.8	12.8
	Nb-C-Nb	2.7	2.74	1.8	
	Nb-Nb	0.2	3.17	0.3	
Pd/Br-Nb_2C	Nb-C	2.1	2.12	1.1	10.9
	Nb-C-Nb	4.4	2.79	2.3	
	Nb-Nb	0.5	3.16	0.3	

[a]CN is the coordination number; [b]R is interatomic distance; [c]σ^2 is Debye-Waller factor (a measure of thermal and static disorder in absorber-scatter distances); S_0^2 was obtained from Nb metal and fixed as 0.80; ΔE_0 shift in absorption edge energy, Data ranges $3.000 \leq k \leq 11$ Å^{-1}, $1.3 \leq R \leq 3.1$ Å. The number of variable parameters is 3, R factor for these fits are 0.6%, 0.9%, 1.9%.

Supplementary Table 4. Full elementary steps for CHCR hydrogenation.

Elementary Reaction Steps
(r1) H_2+*+*→H*+H*
(r2) CHCR+*→CHCR*
(r3) CHCR*+H*→CHCHR*+*
(r4) CHCR*+H*→$CRCH_2$*+*
(r5) CHCHR*+H*→CH_2CHR*+*
(r6) $CRCH_2$*+H*→CH_2CHR*+*
(r7) CH_2CHR*→CH_2CHR+*
(r8) CHCHR*+H*→$CHCH_2R$*+*
(r9) $CRCH_2$*+H*→$CRCH_3$*+*
(r10) CH_2CHR*+H*→CH_2CH_2R*+*
(r11) CH_2CHR*+H*→$CHRCH_3$*+*
(r12) CH_2CH_2R*+H*→CH_3CH_2R*+*
(r13) $CHRCH_3$*+H*→CH_3CH_2R*+*
(r14) CH_3CH_2R*→CH_3CH_2R+*
(r15) $CHCH_2R$*+H*→CH_2CH_2R*+*
(r16) $CRCH_3$*+H*→$CHRCH_3$*+*

Supplementary Table 5. The barriers and reaction energies of elementary reactions on Pd (111) for the hydrogenation phenylacetylene without the coverage effect. All elementary reaction steps include free energy corrections. (T=298.15 K)

Elementary reaction	Forward reaction barrier (eV)	Reaction Energy (eV)
H_2+*+*→H*+H*	0.00	-1.10
CHCR+*→CHCR*	0.00	-3.13
CHCR*+H*→CHCHR*+*	1.13	-0.08
CHCR*+H*→$CRCH_2$*+*	0.99	0.13
CHCHR*+H*→CH_2CHR*+*	0.84	-0.08
$CRCH_2$*+H*→CH_2CHR*+*	0.80	-0.29
CH_2CHR*→CH_2CHR+*	2.77	2.77
CHCHR*+H*→$CHCH_2R$*+*	1.24	0.48
$CRCH_2$*+H*→$CRCH_3$*+*	0.88	0.31
CH_2CHR*+H*→CH_2CH_2R*+*	1.29	0.79
CH_2CHR*+H*→$CHRCH_3$*+*	0.96	0.44
CH_2CH_2R*+H*→CH_3CH_2R*+*	0.89	0.27
$CHRCH_3$*+H*→CH_3CH_2R*+*	0.99	0.62
CH_3CH_2R*→CH_3CH_2R+*	1.97	1.97
$CHCH_2R$*+H*→CH_2CH_2R*+*	0.94	0.23
$CRCH_3$*+H*→$CHRCH_3$*+*	1.01	-0.16

Supplementary Table 6. The barriers and reaction energies of elementary reactions on Pd/Nb_2C for the hydrogenation phenylacetylene without the coverage effect. All elementary reaction steps include free energy corrections. (T=298.15 K)

Elementary reaction	Forward reaction barrier (eV)	Reaction Energy (eV)
H_2+*+*→H*+H*	0.00	-1.11
CHCR+*→CHCR*	0.00	-2.73
CHCR*+H*→CHCHR*+*	1.15	0.13
CHCR*+H*→$CRCH_2$*+*	0.99	0.56
CHCHR*+H*→CH_2CHR*+*	0.88	-0.04
$CRCH_2$*+H*→CH_2CHR*+*	0.82	-0.46
CH_2CHR*→CH_2CHR+*	2.11	2.11
CHCHR*+H*→$CHCH_2R$*+*	1.16	0.64
$CRCH_2$*+H*→$CRCH_3$*+*	1.06	0.06
CH_2CHR*+H*→CH_2CH_2R*+*	1.25	0.96
CH_2CHR*+H*→$CHRCH_3$*+*	0.84	0.39
CH_2CH_2R*+H*→CH_3CH_2R*+*	0.63	0.25
$CHRCH_3$*+H*→CH_3CH_2R*+*	0.83	0.82
CH_3CH_2R*→CH_3CH_2R+*	1.17	1.17
$CHCH_2R$*+H*→CH_2CH_2R*+*	0.90	0.28
$CRCH_3$*+H*→$CHRCH_3$*+*	1.01	-0.13

Supplementary Table 7. The barriers and reaction energies of elementary reactions on Pd (111) for the hydrogenation phenylacetylene with the coverage effect. All elementary reaction steps include free energy corrections. (T=298.15 K)

Elementary reaction	Forward reaction barrier (eV)	Reaction Energy (eV)
H_2+*+*→H*+H*	0.00	-0.52
CHCR+*→CHCR*	0.00	-1.08
CHCR*+H*→CHCHR*+*	0.53	-0.29
CHCR*+H*→$CRCH_2$*+*	0.86	0.13
CHCHR*+H*→CH_2CHR*+*	0.78	-0.29
$CRCH_2$*+H*→CH_2CHR*+*	0.36	-0.70
CH_2CHR*→CH_2CHR+*	0.55	0.55
CHCHR*+H*→$CHCH_2R$*+*	0.62	0.25
$CRCH_2$*+H*→$CRCH_3$*+*	0.73	0.13
CH_2CHR*+H*→CH_2CH_2R*+*	1.34	0.60
CH_2CHR*+H*→$CHRCH_3$*+*	0.58	0.25
CH_2CH_2R*+H*→CH_3CH_2R*+*	0.43	-0.78
$CHRCH_3$*+H*→CH_3CH_2R*+*	0.46	-0.43
CH_3CH_2R*→CH_3CH_2R+*	0.41	0.41
$CHCH_2R$*+H*→CH_2CH_2R*+*	0.48	0.07
$CRCH_3$*+H*→$CHRCH_3$*+*	0.42	-0.58

Supplementary Table 8. The barriers and reaction energies of elementary reactions on Pd/Nb_2C for the hydrogenation phenylacetylene with the coverage effect. All elementary reaction steps include free energy corrections. (T=298.15 K)

Elementary reaction	Forward reaction barrier (eV)	Reaction Energy (eV)
H_2+*+*→H*+H*	0.00	-0.75
CHCR+*→CHCR*	0.00	-0.98
CHCR*+H*→CHCHR*+*	0.14	-0.15
CHCR*+H*→$CRCH_2$*+*	0.88	0.26
CHCHR*+H*→CH_2CHR*+*	0.83	0.27
$CRCH_2$*+H*→CH_2CHR*+*	0.53	-0.14
CH_2CHR*→CH_2CHR+*	0.00	-0.01
CHCHR*+H*→$CHCH_2R$*+*	0.53	0.42
$CRCH_2$*+H*→$CRCH_3$*+*	0.95	0.04
CH_2CHR*+H*→CH_2CH_2R*+*	0.58	0.50
CH_2CHR*+H*→$CHRCH_3$*+*	0.16	0.04
CH_2CH_2R*+H*→CH_3CH_2R*+*	0.29	-0.92
$CHRCH_3$*+H*→CH_3CH_2R*+*	0.27	-0.46
CH_3CH_2R*→CH_3CH_2R+*	0.33	0.33
$CHCH_2R$*+H*→CH_2CH_2R*+*	0.46	0.35
$CRCH_3$*+H*→$CHRCH_3$*+*	0.52	-0.14

Supplementary Table 9. Data and experimental conditions for substrate hydrogenation in Table 1.

Entry	Substrate	Pressure (MPa) /Temperature (K)	Pd /Substrate (mol %)	Time (min)	Conv. (%)	Sel. (%)
1	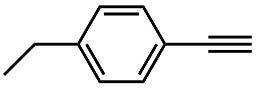	0.1/298	0.023	12	94	95
2	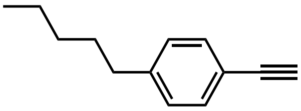	0.1/298	0.023	30	96	92
3	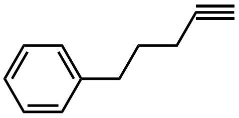	0.1/298	0.023	55	95	94
4	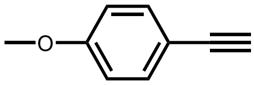	0.1/298	0.023	55	96	94
5	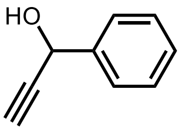	0.2/298	0.023	20	96	93
6	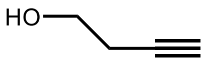	0.1/303	0.047	50	92	93
7	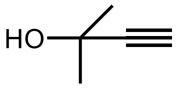	0.2/298	0.023	11	99	92

8	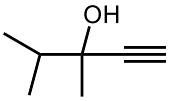	0.2/298	0.023	45	97	92
9	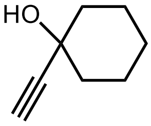	0.1/303	0.023	20	96	91
10	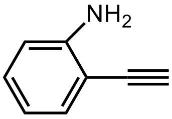	0.1/298	0.023	25	92	95
11	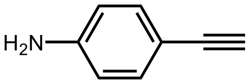	0.1/298	0.023	60	99	93
12	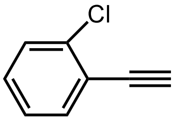	0.2/298	0.023	20	99	91
13	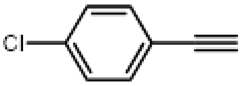	0.2/298	0.023	25	94	92
14	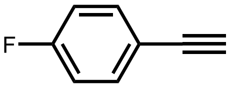	0.1/298	0.023	80	98	93
15	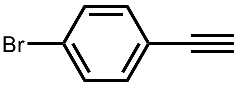	0.1/298	0.023	100	96	93
16	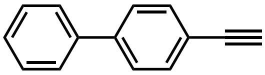	0.1/303	0.023	60	92	95
17	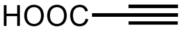	0.1/303	0.023	26	93	92
18	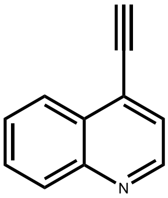	0.1/303	0.047	10	99	96

19	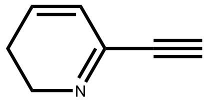	0.1/303	0.047	40	88	89
20	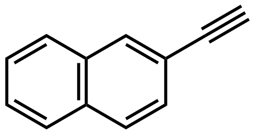	0.1/303	0.115	30	99	88
21	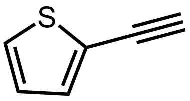	0.1/313	0.023	180	87	94
22	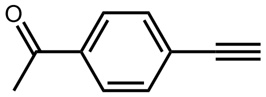	0.1/313	0.047	40	99	88
23	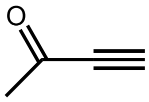	0.1/298	0.023	35	99	94
24	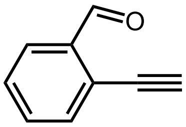	0.1/313	0.047	180	99	93
25	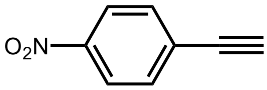	0.1/298	0.047	180	99	92
26	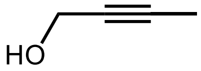	0.2/298	0.023	59	96	96(cis)
27	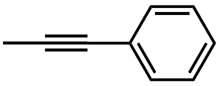	0.2/298	0.023	55	97	96(cis)
28	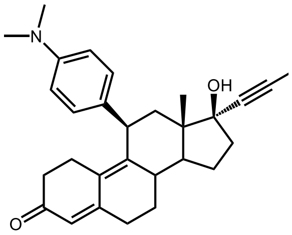	0.2/303	0.200	600	97	90(cis)

These substrates experiments are all used 5 mL ethanol as solvent, Pd/Nb_2C.

Supplementary References

1. Marquardt, D. W. An Algorithm for Least-Squares Estimation of Nonlinear Parameters. *J. Soc. Ind. Appl. Math.* **11**, 431-441 (1963).

2. Lagunas, F. A method for the solution of certain non-linear problems in least squares. *Quar. Appl. Math.* **2**, 164-168 (1944).

3. Plimpton, S. Fast Parallel Algorithms for Short-Range Molecular Dynamics. *J. Comput. Phys.* **117**, 1-19 (1995).

4. Daw, M. S., Baskes, M. I. Embedded-atom method: Derivation and application to impurities, surfaces, and other defects in metals. *Phys. Rev. B* **29**, 6443-6453 (1984).

5. Foiles, S. M., Baskes, M. I., Daw, M. S. Embedded-atom-method functions for the fcc metals Cu, Ag, Au, Ni, Pd, Pt, and their alloys. *Phys. Rev. B* **33**, 7983-7991 (1986).

6. Perdew, J. P., Burke, K., Ernzerhof, M. Generalized Gradient Approximation Made Simple. *Phys. Rev. Lett.* **77**, 3865-3868 (1996).

7. Kresse, G., Furthmüller, J. Efficiency of ab-initio total energy calculations for metals and semiconductors using a plane-wave basis set. *Comput. Mater. Sci.* **6**, 15-50 (1996).

8. Kresse, G., Hafner, J. Ab initio molecular-dynamics simulation of the liquid-metal--amorphous-semiconductor transition in germanium. *Phys. Rev. B* **49**, 14251-14269 (1994).

9. Kresse, G., Joubert, D. From ultrasoft pseudopotentials to the projector augmented-wave method. *Phys. Rev. B* **59**, 1758-1775 (1999).

10. Blöchl, P. E., Jepsen, O., Andersen, O. K. Improved tetrahedron method for Brillouin-zone integrations. *Phys. Rev. B* **49**, 16223-16233 (1994).

11. Wei, Z. Z.*, et al.* Optimizing Alkyne Hydrogenation Performance of Pd on Carbon in Situ Decorated with Oxygen-Deficient TiO2 by Integrating the Reaction and Diffusion. *ACS Catal.* **9**, 10656-10667 (2019).

12. Yao, Z. H., Guo, C. X., Mao, Y., Hu, P. Quantitative Determination of C-C Coupling Mechanisms and Detailed Analyses on the Activity and Selectivity for Fischer-Tropsch Synthesis on Co(0001): Microkinetic Modeling with Coverage Effects. *ACS Catal.* **9**, 5957-5973 (2019).

13. Yao, Z., Zhao, J., Bunting, R. J., Zhao, C., Hu, P., Wang, J. Quantitative Insights into the Reaction Mechanism for the Direct Synthesis of H2O2 over Transition Metals: Coverage-Dependent Microkinetic Modeling. *ACS Catal.* **11**, 1202-1221 (2021).

14. Yao, Z.*, et al.* A first-principles study of reaction mechanism over carbon decorated oxygen-deficient TiO2 supported Pd catalyst in direct synthesis of H2O2. *Chin. J. Chem. Eng.* **31**, 126-134 (2021).

15. Xie, P.*, et al.* Oxo dicopper anchored on carbon nitride for selective oxidation of methane. *Nat. Commun.* **13**, 1375 (2022).

16. Klimeš, J., Bowler, D. R., Michaelides, A. Van der Waals density functionals applied to solids. *Phys. Rev. B* **83**, (2011).

17. Grimme, S., Antony, J., Ehrlich, S., Krieg, H. A consistent and accurate ab initio parametrization of density functional dispersion correction (DFT-D) for the 94 elements H-Pu. *J. Chem. Phys.* **132**, 154104 (2010).

18. Grimme, S., Ehrlich, S., Goerigk, L. Effect of the damping function in dispersion corrected density functional theory. *J. Comput. Chem.* **32**, 1456-1465 (2011).

19. Schroder, H., Creon, A., Schwabe, T. Reformulation of the D3(Becke-Johnson) Dispersion Correction without Resorting to Higher than C(6) Dispersion Coefficients. *J. Chem. Theory Comput.* **11**, 3163-3170 (2015).

20. Michaelides, A., Liu, Z. P., Zhang, C. J., Alavi, A., King, D. A., Hu, P. Identification of General Linear Relationships between Activation Energies and Enthalpy Changes for Dissociation Reactions at Surfaces. *J. Am. Chem. Soc.* **125**, 3704-3705 (2003).

21. Liu, Z.-P., Hu, P. General Rules for Predicting Where a Catalytic Reaction Should Occur on Metal Surfaces: A Density Functional Theory Study of C−H and C−O Bond Breaking/Making on Flat, Stepped, and Kinked Metal Surfaces. *J. Am. Chem. Soc.* **125**, 1958-1967 (2003).

22. Zhang, C., Hu, P., Alavi, A. A General Mechanism for CO Oxidation on Close-Packed Transition Metal Surfaces. *J. Am. Chem. Soc.* **121**, 7931-7932 (1999).

23. Alavi, A., Hu, P., Deutsch, T., Silvestrelli, P. L., Hutter, J. CO Oxidation on Pt(111): An Ab Initio Density Functional Theory Study. *Phys. Rev. Lett.* **80**, 3650-3653 (1998).

24. Cortright, R. D., Dumesic, J. A. Kinetics of heterogeneous catalytic reactions:

Analysis of reaction schemes. *Adv. Catal.* **46**, 161-264 (2001).
