# Supplementary material for: Tripodal Pd metallenes mediated by Nb2C MXenes for boosting alkynes semihydrogenation
Source: Nat Commun. 2023 Feb 7;14:661. doi: 10.1038/s41467-023-36378-3 (PMC9905561; doi:10.1038/s41467-023-36378-3)
Supplement: Supplementary file 3 — Description of Additional Supplementary Files [file 41467_2023_36378_MOESM3_ESM.pdf]

Description of Additional Supplementary Files

File name: Supplementary Movie 1
Description: The structure evolution process of Pd nanoclusters on Nb_2C modified with oxygen atoms at the top view.

File name: Supplementary Movie 2
The structure evolution process of Pd nanoclusters on Nb_2C modified with oxygen atoms at the side view.

File name: Supplementary Movie 3
The structure evolution process of Pd nanoclusters on Nb_2C modified with oxygen atoms at the front view.
